# Socioeconomic and demographic characterization of an endemic malaria region in Brazil by multiple correspondence analysis

**DOI:** 10.1186/s12936-017-2045-z

**Published:** 2017-10-02

**Authors:** Raquel M. Lana, Thais I. S. Riback, Tiago F. M. Lima, Mônica da Silva-Nunes, Oswaldo G. Cruz, Francisco G. S. Oliveira, Gilberto G. Moresco, Nildimar A. Honório, Cláudia T. Codeço

**Affiliations:** 1 Programa de Pós-Graduação em Epidemilogia em Saúde Pública, Escola Nacional de Saúde Pública Sérgio Arouca, Fundação Oswaldo Cruz, Rua Leopoldo Bulhões, 1480, Manguinhos, Rio de Janeiro, RJ 21041-210 Brazil; 2 Programa de Computação Científica, Fundação Oswaldo Cruz, Residência Oficial, Avenida Brasil, 4365, Manguinhos, Rio de Janeiro, RJ 21040-360 Brazil; 3 Laboratório de Engenharia e Desenvolvimento de Sistemas, Departamento de Computação e Sistemas, Instituto de Ciências Exatas e Aplicadas, Universidade Federal de Ouro Preto., Rua 36, n. 115, Loanda, João Monlevade, MG 35931-008 Brazil; 4 Centro de Ciências da Saúde, Universidade Federal do Acre, Campus Universitário-BR 364, km 4-Distrito Industrial, Rio Branco, AC 69920-900 Brazil; 5 Campus Cruzeiro do Sul, Universidade Federal do Acre, Estrada do Canela Fina, s/n, Cruzeiro do Sul, AC 69980-000 Brazil; 6 Coordenação Geral dos Programas Nacionais de Controle e Prevenção da Malária e das Doenças transmitidas pelo Aedes, Departamento de Vigilância das Doenças Transmissíveis, Secretaria de Vigilância em Saúde-Ministério da Saúde, SRTV 702, Via W 5 Norte, Ed. PO700-6 andar, Brasília, DF 70723-040 Brazil; 7 Laboratório de Mosquitos Transmissores de Hematozoários-Lathema, Instituto Oswaldo Cruz, FIOCRUZ, Avenida Brasil, 4365, Manguinhos, Rio de Janeiro, RJ 21040-360 Brazil; 8 Núcleo Operacional Sentinela de Mosquitos Vetores-Nosmove/FIOCRUZ, Avenida Brasil, 4365, Manguinhos, Rio de Janeiro, RJ 21040-360 Brazil

**Keywords:** Urban malaria, Rurality, Multiple correspondence analysis, Amazon, Micro-epidemiology

## Abstract

**Background:**

In the process of geographical retraction of malaria, some important endemicity pockets remain. Here, we report results from a study developed to obtain detailed community data from an important malaria hotspot in Latin America (Alto Juruá, Acre, Brazil), to investigate the association of malaria with socioeconomic, demographic and living conditions.

**Methods:**

A household survey was conducted in 40 localities (n = 520) of Mâncio Lima and Rodrigues Alves municipalities, Acre state. Information on previous malaria, schooling, age, gender, income, occupation, household structure, habits and behaviors related to malaria exposure was collected. Multiple correspondence analysis (MCA) was applied to characterize similarities between households and identify gradients. The association of these gradients with malaria was assessed using regression.

**Results:**

The first three dimensions of MCA accounted for almost 50% of the variability between households. The first dimension defined an urban/rurality gradient, where urbanization was associated with the presence of roads, basic services as garbage collection, water treatment, power grid energy, and less contact with the forest. There is a significant association between this axis and the probability of malaria at the household level, OR = 1.92 (1.23–3.02). The second dimension described a gradient from rural settlements in agricultural areas to those in forested areas. Access via dirt road or river, access to electricity power-grid services and aquaculture were important variables. Malaria was at lower risk at the forested area, OR = 0.55 (1.23–1.12). The third axis detected intraurban differences and did not correlate with malaria.

**Conclusions:**

Living conditions in the study area are strongly geographically structured. Although malaria is found throughout all the landscapes, household traits can explain part of the variation found in the odds of having malaria. It is expected these results stimulate further discussions on modelling approaches targeting a more systemic and multi-level view of malaria dynamics.

**Electronic supplementary material:**

The online version of this article (doi:10.1186/s12936-017-2045-z) contains supplementary material, which is available to authorized users.

## Background

The American continent witnessed a large reduction in malaria incidence in the last century, with 18 out of 21 previously malaria-endemic countries now close to elimination [1]. Malaria activity is currently concentrated in the Amazonian region (including Brazil, Colombia, Peru, and Venezuela) where *Plasmodium falciparum* and *Plasmodium vivax* are the two main parasites. In Brazil, morbidity and mortality by falciparum malaria decreased since the 1980’s [2] and, in 2010, it was mostly restricted to the Northwest Amazon, while more than 80% of malaria burden in the Brazilian and Peruvian Amazon [2, 3], and 67% in Colômbia [4] is attributed to *P. vivax*. Controlling vivax malaria is more challenging for several reasons: the large proportion of asymptomatic and sub-microscopic infections are more difficult to detect and cure; and its latent stage, lasting weeks to months, is refractory to standard treatments [5]. Relapses from latent infections account for 14–40% of malaria episodes in the Amazon [6].

Despite low mortality, vivax malaria imposes a high disease burden in malaria transmission hotspots, where individuals can experience several debilitating episodes per year, impairing their capacity to work and study. Although not frequent, *P. vivax* infection is associated with unusual complications [7]. During pregnancy, it is a cause of stillbirth and low birth weight, and during infancy, it may affect child development [5].

Subsequent waves of colonization and development in the Amazon have created strong spatial heterogeneities, with the coexistence of small and scattered communities along rivers, agricultural settlements along “fishbone” road networks, and towns with different levels of development [8]. These communities are connected by the flow of individuals seeking health, banking services, purchasing goods, attending school and working [9]. The population along rivers are composed mostly of indigenous and descendants of immigrants from the nineteenth and mid-twentieth centuries entering the rubber industry. In the 1940’s, a large wave of colonization driven by rubber exploitation populated many of the larger localities found today. More recently, immigrants were attracted to the settlements created by the National Institute for Land Reform (INCRA) along new roads crossing the Amazonian region. These parallel secondary roads form “a fishbone land occupation scheme” that can easily be seen from satellite, creating an extended deforestation fringe. Within the Brazilian Amazon, three municipalities (Mâncio Lima, Rodrigues Alves and Cruzeiro do Sul) constitute a persistent malaria hotspot (defined as a site with significantly higher disease risk than average), for both *P. falciparum* and *P. vivax* parasites [10]. Here, this area will be referred to as the “Juruá hotspot” in reference to the administrative region where it is located. Many studies have assessed large-scale drivers of malaria in the Amazon, and more specifically, in the Juruá hotspot, associating malaria risk with forest cover [10], deforestation [11], and fish farming [12]. Malaria transmission in this area is found in both rural and urban zones, and the Juruá hotspot constitutes one of the seven sites for urban malaria studies of the International Centers of Excellence for Malaria Research (ICEMR) [13].

Micro-epidemiology is the study of fine-scale variations in risk? between households or other sub-village groupings within villages, or between neighbouring villages or other similar socio-spatial aggregations such as urban neighbourhoods, agricultural settlements and health centre catchment areas? [14]. The household survey described here is a micro-epidemiology study conducted in the Juruá hotspot to investigate the distribution of malaria and its interaction with socio-economic, behavioural and demographic parameters along gradients of development. Using multivariate analysis, living conditions associated with exposure to malaria were identified, providing a characterization of this hard-to-reach population at a geographical scale not studied before. As this population is under strong environmental and development change, this study provides a baseline for subsequent monitoring activities and guide the application of interventions.

## Methods

### Study area

The study area is located within two municipalities in the Alto Juruá region, Acre state, Brazil. Mâncio Lima (ML) (7.5468${^\circ }$S, 73.3709$^{\circ }$W) has 15,206 inhabitants (2.79 inhabitants/km$^{2}$), and Rodrigues Alves (RA) (7.8819$^{\circ }$S, 73.3709$^{\circ }$W) has 14,389 inhabitants (4.68 inhabitants/km$^{2}$) [15]. Their administrative centers are connected to each other (40 km) and to Cruzeiro do Sul (CS) by a paved road (CS-RA: 12 km, CS-ML: 43 km). CS is Acre’s second-largest city (78,507 inhabitants), has an airport with daily flights to the state capital, Rio Branco. A paved 700 km road connects CS to Rio Branco. These modern modes of transportation coexist with the traditional and heavily used waterway transport (the Juruá River is the main commodity transportation route between the neighbour municipalities of the Alto Juruá region and Manaus, in the Amazon as state). Two-thirds of ML is occupied by protected areas, including the Serra do Divisor National Park and indigenous reserves. About half (57.3%) of the Mâncio Lima population lives in the administrative center (a town divided into 9 localities or neighbourhoods), the remainder distributed in 57 small localities scattered along dirt roads and rivers. In Rodrigues Alves (RA), 30% lives in a small town (5 localities or neighbourhoods) and 70% in 69 rural settlements.

### Household survey

**Fig. 1:**
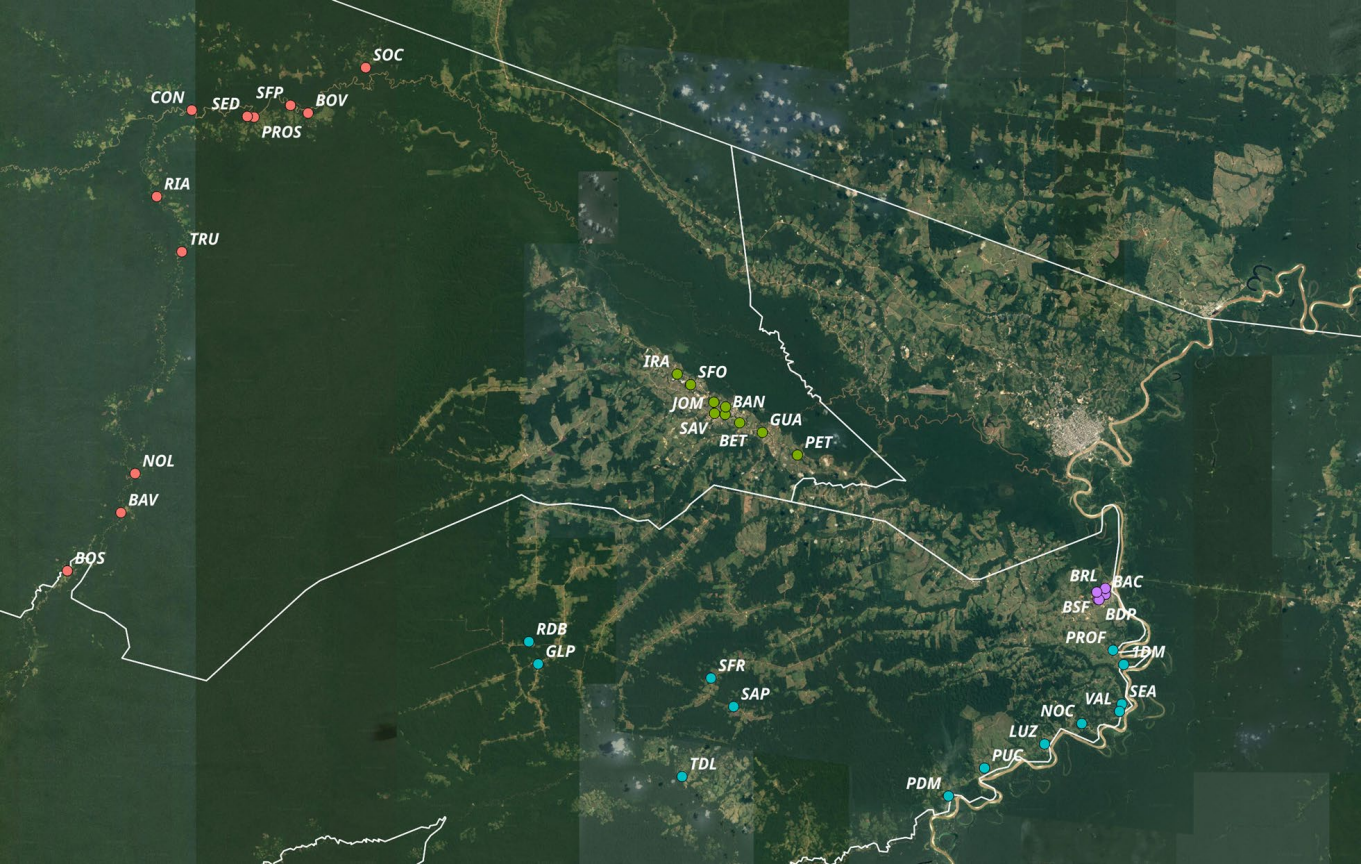
Map of the municipalities of Mâncio Lima and Rodrigues Alves, Acre, Brazil, with the 40 localities included in the survey. The point labels are abbreviations of the localities’ names (full names in Additional file 1). Points are colored according to the different zones: ML-r (red), ML-u (green), RA-r (blue) and RA-u (violet). Source of image: Google MAPS API (https://developers.google.com/maps/terms), 2017. Software QGis Version 2.18

The survey, conducted in 2015, included 40 localities, being 5 in the town of Mâncio Lima (ML-u), 9 in the town of Rodrigues Alves (RA-u), 13 in the rural area of Mâncio Lima (ML-r) and 13 in the rural area of Rodrigues Alves (RA-r). ML-r was surveyed in February when river level was high, the two towns (ML-u and RA-u) were surveyed in May, and the road accessible rural localities in RA-r were surveyed in July, during the dry season when roads are open (Fig. 1).

#### Riverine localities (ML-r)

ML-r localities are located along the Moa river and its tributary, the Azul river. They are accessible only by boat, taking up to 2 days to reach the farthest point during the rainy season. The landscape is mostly covered by forest; the population lives mainly by manioc production, fishing and, social welfare. From the total of 26 localities, 20 non-indigenous, 13 were surveyed (66%). Outdated official data indicated 399 households in the 13 localities but 182 households were found inquiring the local population, varying from 3 to 40 households per locality. For logistic reasons, the sampling effort was concentrated at the core of each locality, excluding isolated single households and not returning to those that were closed. A total of 107 households were interviewed in this area (sample effort = 58.7%), during the 6 days of the survey, corresponding to 505 dwellers.

#### Localities in the two towns (ML-u and RA-u)

All 15 within-town localities were included in the survey. The number of households in ML-u was 2589, varying from 32 to 730 per locality. RA-u had 1497 households, varying from 160 to 389 households per locality. A systematic sampling scheme was adapted to the geography of each town. In ML, every 8th to 12th house along a predefined route was surveyed, in a protocol similar to the Census, totaling 190 households (sample size = 7.3%) with 732 dwellers. In RA, 102 households (with 459 dwellers) were sampled, by choosing a side from each of the 102 lots and randomly sampling 1 household (sample size = 6.8%).

#### Rural localities accessible by road (RA-r)

RA-r localities are agricultural settlements distributed along 3 dirt roads radiating from the ML-RA main road, an example of a fishbone colonization pattern. The landscape is dominated by pastures but also includes areas of recent deforestation. During the monsoons, road access is mostly limited to 4 × 4 vehicles and motorcycles and may be completely blocked for weeks. Alternative access to some localities is possible via the Juruá river and its tributaries. The 13 surveyed localities varied in size from 18 to 133 households, totaling 2728 households. At each locality, the survey team sketched a map and selected houses the most evenly possible. The final sample size was 121 households (4.4%), with 578 dwellers.

The complete list of surveyed localities is found in Additional file 1, and representative images are found in Additional file 2.

### Questionnaire

The questionnaire was adapted from [16] to include questions that are specific to rural settings, such as the ownership of farm animals, as well as blocks covering mobility, access to health care, and behaviors associated with malaria exposure. A pilot study was conducted before the survey to test the questionnaire for clarity. The questionnaire is divided into 17 blocks of which, 14 are included in the present study (Table 1). Blocks A and B collect demographic information from the interviewee and the house mates, blocks C, D and E inquiry about their history of malaria infections, block F records the ownership of objects such as appliances and vehicles, block G records all the occupations and sources of income of the householders. Five blocks collect data on house construction material (H), accessibility (I), source of water (J), garbage disposal (L), and electricity (M). Questions on activities and habits associated with malaria exposure and usage of bed nets as well as other mosquito deterrents are in the last blocks (N, O). The questionnaire was answered by a householder, 18 years of age or more, who could respond for the other householders and agreed with the study, signing the informed consent. The research protocol was approved by *Comitê de Ética da Escola Nacional de Saúde Pública Sérgio Arouca* (Ethics Committee of the National School of Public Health Sérgio Arouca) (ENSP-Fiocruz), in 04/11/2014, Number 861.871.

**Table 1:** Questionnaire description

Block	Information
(A) Interviewee demographic data	Name
	Address
	Birth date and age
	Gender
	Race/color
	Birth place
	Residence time in the current address
(B) Dwellers demographic data	Name
	Age
	Gender
	Relationship with the interviewee
	Residence time > 12 months in the address
	Schooling
	School’s name and location
(C) Malaria episodes reported by the interviewee	If remembers the 1st malaria
	Self-reported malaria in the last 12 months
	Inactivity and days lost due to malaria
	If needed health care in the last 12 months
	Places sought for health care
	Hospitalization due to malaria
(D) Interviewee’s last episode of malaria	How he/she discovered he/she had malaria
	Date of the last malaria episode (self-report)
	If he/she had malaria symptoms
	Type of malaria (falc, vivax)
	Access and adherence to treatment
(E) Malaria information for each dweller	If they had malaria in the last 12 months (as reported by the interviewee)
	Hospitalization to treat malaria
(F) Household goods	Ownership of goods and animals
(G) Occupation and income of the household	Types of occupation of the householders
	Number of dwellers contributing for the household income
	If the income received is considered sufficient
(H) Characteristics of household	Material used for the wall, roof, ceiling and floor
	Presence of crevices in the walls
	Number of rooms
(I) Household access	Type of access to the household (river, road, trail)
	If household is accessible by vehicle on a rainy day
	If access is paved
	If you would like the street, road or trail to be paved
	If the access to the house floods
	If flooding reaches the door of the house
	Presence of natural or artificial water bodies in the vicinity of the house and the neighborhood
(J) Water supply	Type of access to water
	Source of water used in the kitchen and for drinking
	How water is stored
	If water is treated before consumption
	Frequency at which water is missing at home
	Place where the households shower
	Place where the households wash dishes and clothes
	Place where the households go to tend to their physiological needs
(L) Garbage disposal	How waste is disposed
	Frequency of garbage collection by public service, if available
(M) Access to electricity	If there is electricity in the household
	If available: its source, installation date, if time-restricted or fails, cost
	If not available: source of energy used
(N) Mobility and potential exposure to malaria	Routine activities from 6 p.m. to 9 p.m.
	Frequency and motivation to go close to water bodies and the forest
	Frequency of sleeping in the wild
	Frequency, motivation, and length of stay in farms
	If needs to travel to receive social benefits, frequency and length of stay
	If needs to travel for working, frequency and length of stay
	If traveled for any other reason in the last 12 months, frequency and length of stay
(O) Use of bednet and other protections	Ownership of impregnated and standard bednets and quantity
	General usage, usage in the last night
	Problems with bednet usage
	If the households use other methods for mosquito protection
	Recommendations or suggestions for improving the health of the family and community

### Statistical methods

#### Variables

Analysis was carried out at the household level. Individual level variables (age, gender, schooling) were aggregated as following: *children* (presence of dwellers < 14 years old), *elderly* (presence of dwellers ≥ 60 years old), *adult gender* (only women, only men or both), *adult gender 1* (uni or pluri gender), *maximum schooling* (with 9 categories), *maximum schooling 1* (with 5 categories), *illiteracy in dwellers aged* ≥ *18 years 1* (0, ≥ 1), *illiteracy in dwellers aged* ≥ *18 years 2* (0, 1 or 2), *illiteracy in dwellers aged* ≥ *18 and* ≤ *59* (0, 1 or 2), *illiteracy in dwellers aged* ≥ *60 years* (0, 1 or 2). Information on previous malaria episodes was used to construct the variable *household with malaria in the last 12 months* (POS/NEG). Before proceeding, a preliminary analysis was carried out to identify and correct ill-formed variables. For example, variables with categories with fewer than 5 elements were recategorized; if two variables belonging to the same block were strongly correlated, one of them was chosen or a combination of both was created.

#### Multiple correspondence analysis (MCA)

**Fig. 2:**
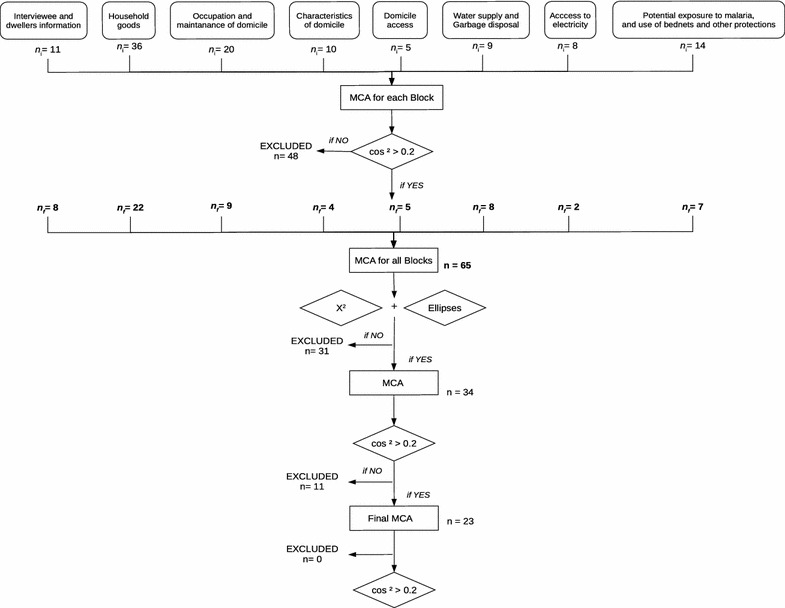
Flowchart describing the steps for the construction of the MCA factor map

MCA is a multivariate exploratory analysis for visualizing large datasets of categorical variables [17, 18]. Its graphical visualization provides a structural organization for the variables and categories in a dimensional space that is useful for identifying patterns in the data and associations between the investigated parameters [18]. Here, MCA was used to elicit gradients of living conditions in the Juruá malaria hotspot. To carried out the analysis, variables were divided into 8 groups (Table 2). Malaria in the last 12 months (yes/no) was included as a supplementary variable. Supplementary variables do not contribute to the MCA but can be plotted together to provide useful visualization, in this case, of the distribution of malaria along the development gradients [19]. The MCA map was constructed step wisely (Fig. 2). For each of the 8 groups of variables, the MCA was applied and variables were selected using the squared cosine test. All variables with $cos^{2}$ > 0.2 in at least one of the 3 first MCA dimensions were maintained in this first round. Some variables, such as ‘presence of swamps’, did not meet this criterion but were maintained due to their known association with malaria. In some instances, before excluding a variable, different categorizations were attempted and a variable was discarded only if no alternative version attended the selection criterion. Variables with missing data can strongly distort MCA results, so after comparing MCA with and without these variables, those with strong leverage effect were removed. The next step was to merge all selected variables into a single group and apply the MCA. Three rounds of MCA were performed in order to remove non-significant variables. In the first round, confidence ellipses were used for identifying nonsignificant categories that were collapsed into single categories [20]. This procedure helped to reduce the number of categories per variable. Second, the association between each variable and the response variable *Household with or without malaria in the last 12 months* was tested using chi-squared contingency test [20]. When confidence ellipses of all categories of a variable included the origin of the MCA plot and the chi-squared test was not significant (at p $\le$ 0.2), the variable was excluded. However, some variables considered important from the theoretical perspective, such as those associated with malaria exposure (e.g., owning a boat) or a well-established indicator of income (owning a fridge) were maintained independently of these selection criteria. The same procedure was repeated in the second and third rounds, restricting to the application of the $cos^{2}$ rule (Fig. 2). The number of dimensions maintained in the final model was determined based on their inertia, that is, the percentage of variance explained.

**Table 2:** Summary of the variables and its categories, organized by block, and the result of the chi-squared test for differences in their distribution between study zones (ML-u, ML-r, RA-u, RA-r) and between households with and without malaria in the last 12 months

Block description	Variables	Category	n	$X^{2}$Zone	$X^{2}$Malaria
Dwellers demographics (AB)	Study zone	Rural Mâncio Lima	107	RV	++
		Urban Mâncio Lima	190		
		Rural Rodrigues Alves	121		
		Urban Rodrigues Alves	102		
	Maximum schooling^a^	Illiterate	19	++	+
		Incomplete middle school	144		
		Complete middle school + incomplete high school	95		
		Complete high school	173		
		Incomplete or complete undergraduation	86		
	Adult illiteracy ≥ 18 years old	None	404	++	+
		At least one	116		
	Adult illiteracy ≥ 18 years old	None	404	++	++
		One	86		
		Two	30		
	Adult illiteracy > 18 and < 60 years^a^	None	448	++	++
		One	61		
		Two	11		
	Adult illiteracy ≥ 60 years old	None	467	−	−
		One	43		
		Two	10		
	Gender in adults	Heterogeneous = men and women	443	+	++
		Homogeneous = only one gender	77		
	Child	None	176	++	+
		At least one	344		
	Elderly	None	409	−	−
		At least one	111		
Malaria information for the interviewed and dwellers(CDE)	Report of malaria in the last 12 months	None	287	++	RV
		At least one	233		
Household goods (F)	Stove	Yes	492	++	−
		No	28		
	Refrigerator	Yes	433	++	−
		No	87		
	Television^b^	Yes	461	++	−
		No	59		
	Washing machine^b^	Yes	305	++	+
		No	215		
	Iron^b^	Yes	177	++	+
		No	343		
	Sofa	Yes	344	++	−
		No	176		
	Blender^b^	Yes	340	++	+
		No	180		
	Video tape or dvd	Yes	234	++	−
		No	286		
	Microwave	Yes	56	++	++
		No	464		
	Car	Yes	56	++	−
		No	464		
	Motorcycle	Yes	194	++	−
		No	326		
	Motor boat^b^	Yes	184	++	−
		No	336		
	Horse	Yes	47	++	++
		No	473		
	Ox^b^	Yes	126	++	−
		No	394		
	Chicken	Yes	335	++	++
		No	185		
	Pig	Yes	74	++	−
		No	446		
	Chainsaw	Yes	115	++	+
		No	405		
	Mobile phone type smartphone	Yes	141	++	−
		No	379		
	Internet on the mobile phone	Yes	121	++	−
		No	399		
	Facebook	Yes	68	++	−
		No	452		
	Fishpond^a,b^	Presence of reservoir with or without fish	49	++	+
		None	471		
	If has computer and if has internet on the computer	None	441	++	−
		Only computer	34		
		Both	45		
Occupation and maintenance of the household (G)	Dweller working in agriculture^b^	Yes	170	++	++
		No	350		
	Works fishing or sailing fish	Yes	47	++	−
		No	473		
	Sells products collected from the forest	Yes	6	−	−
		No	514		
	Works in market	Yes	14	+	−
		No	506		
	Government employee	Yes	73	++	−
		No	447		
	Works with formal contract	Yes	39	++	−
		No	481		
	Driver or boatman	Yes	12	+	−
		No	508		
	Social benefits as Bolsa Familia, Bolsa Verde, Bolsa Pesca and payments	Yes	303	++	+
		No	217		
	Labor benefits as: retirement, health assistance, unemployment benefits and pensioner	Yes	158	++	−
		No	362		
Characteristics of the household (H)	If the house has or not ceiling lining^b^	Throughout the household	53	++	+
		In part of the household	81		
		Do not have	386		
	Type of house floor	Cement or ceramics	187	++	−
		Wood	333		
	Cracks in the house^b^	It has cracks	397	++	−
		It has not cracks	123		
	Wall material^b^	Brick or brickwork	99	++	+
		Wood	421		
Household access (I)	Locality access^a,b^	Access by river	108	++	++
		Paved road	270		
		Dirt road, passage by trail or pathway	142		
	Traffic of some motor vehicle by the road when is raining^b^	Do not have road	112	++	+
		None	37		
		Some vehicle pass through	371		
	If the road or access to the house used to flood^a,b^	Floods	114	++	−
		Do not flood	406		
	If the flood reach the door house	Yes	62	++	−
		No	458		
	If there is a swamp or a small river close to the house	Yes	432	++	−
		No	88		
Water supply and garbage disposal	If there is piped water in the household^b^	Do not have	161	++	−
		Outside the household	201		
		Inside the household	158		
	If the water used to drink or cook in the house came from the public service^b^	Yes	260	++	++
		No	260		
	If the water used to drink or cook in the house came from natural water reservoirs^b^	Yes	147	++	+
		No	373		
	Place of bathing is open or closed	Water bodies or external area of the household	156	++	−
		Bathroom or closed place	364		
	Place for physiological needs^b^	Toilet	215	++	+
		Cesspool or septic tank	246		
		Open place type backyard or forest	59		
	Place for washing the dishes is open or closed^b^	Water bodies or external area of the household	331	++	++
		Inside household	189		
	Garbage is inappropriately discarded^b^	Yes	283	++	++
		No	237		
	Garbage collected by public service^b^	Yes	271	++	++
		No	249		
Access to electricity (M)	It has electricity in the house	Yes	483	++	−
		No	37		
	Type of electricity in the house^b^	Do not have	37	++	+
		Electricity by generator	72		
		Electrical network	411		
Mobility and potential exposure to malaria (NO)	Frequency which reach water bodies and/or forest^a,b^	Rarely or never	208	++	++
		Weekly, once or twice a week	143		
		Daily, usually they are riverine population	169		
	Frequency which sleeps exposed in forests, close to the river, etc.	Rarely or never	414	++	++
		Monthly, once or twice a month	73		
		Weekly, once or twice a week	24		
		Daily	9		
	Frequency which takes recreation bathe	Never	355	++	+
		Sometime, monthly or less	30		
		Probably every month or weekly	24		
		Every day, in general, they are the riverine population	111		
	Frequency which is used to fish or take products on the forest	Never	239	++	−
		Sometime, probably monthly or less	263		
		Probably every month or weekly	4		
		Every day	14		
	Displacement to fetch social benefits or payments^b^	Yes	258	++	++
		No	262		
	There is bednet in the house	Yes	486	++	+
		No	34		
	Never fails to use bednet	Every day, without exception	307	++	−
		Not regularly	213		

*RV* response variable or supplementary variable

^a^Recategorized variables

^b^Variables in the final model

#### Mixed logistic regression

To map the probability distribution of malaria along the MCA gradients, a mixed logistic regression model was fitted. The model has locality as a random intercept to control for clustering of households within localities and a fixed effect for each MCA dimension. Let $Y_{ij}$ = report of malaria in the last 12 months in household *i*, at locality *j*. The model is:

$$\begin{aligned} logit(E(Y_{ij})) = b_{0} + b_{1} * dim1 + b_{2} * dim2 + b_{3} * dim3 + a_{j} \end{aligned}$$

where $b_{0}$ is the intercept, $b_{1}$, $b_{2}$ and $b_{3}$ are the fixed effects of the first, second and third MCA dimensions, and $a_{j}$ is the random intercept with distribution $a_{j} \sim N(0, \sigma ^{2})$. Preliminary analysis using additive models confirmed the linearity of the effect of the covariates on the response variable. All analysis were conducted in R 3.4.1 [21] using the FactoMineR package [20] and the lme4 package [22].

## Results

### Characteristics of the surveyed households

The survey included 520 households totaling 2274 dwellers. Of these, 1112 (48.9%) were female and 1162 (51.1%) male. The mean age was 25.5 years old. A total of 442 (19.9%) persons were reported to have malaria in the last 12 months, 104 (23.53%) were interviewees, and 338 their housemates. At household level, 233 (44.8%) reported at least one episode of malaria in the last 12 months, distributed as: 56 households (10.77%) in ML-r, 80 (15.38%) in ML-u, 66 (12.69%) in RA-r, and 31 (5.96%) in RA-u (p < 0.01). Table 2 shows the Chi-squared association between surveyed variables and the report of malaria by the household. The variables most associated were adult gender, adult illiteracy, ownership of microwave, horse and chicken, working in agriculture, access to the household (river, road), piped water, dishwashing at the river bank, garbage collection, frequency of activities close to rivers and forest (Table 2).

### Gradients of development

**Fig. 3:**
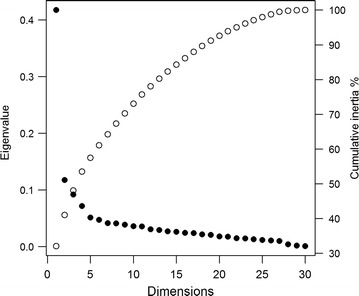
Plot of the MCA eigenvalues (black circles) and cumulative percent of inertia (white circles)

**Fig. 4:**
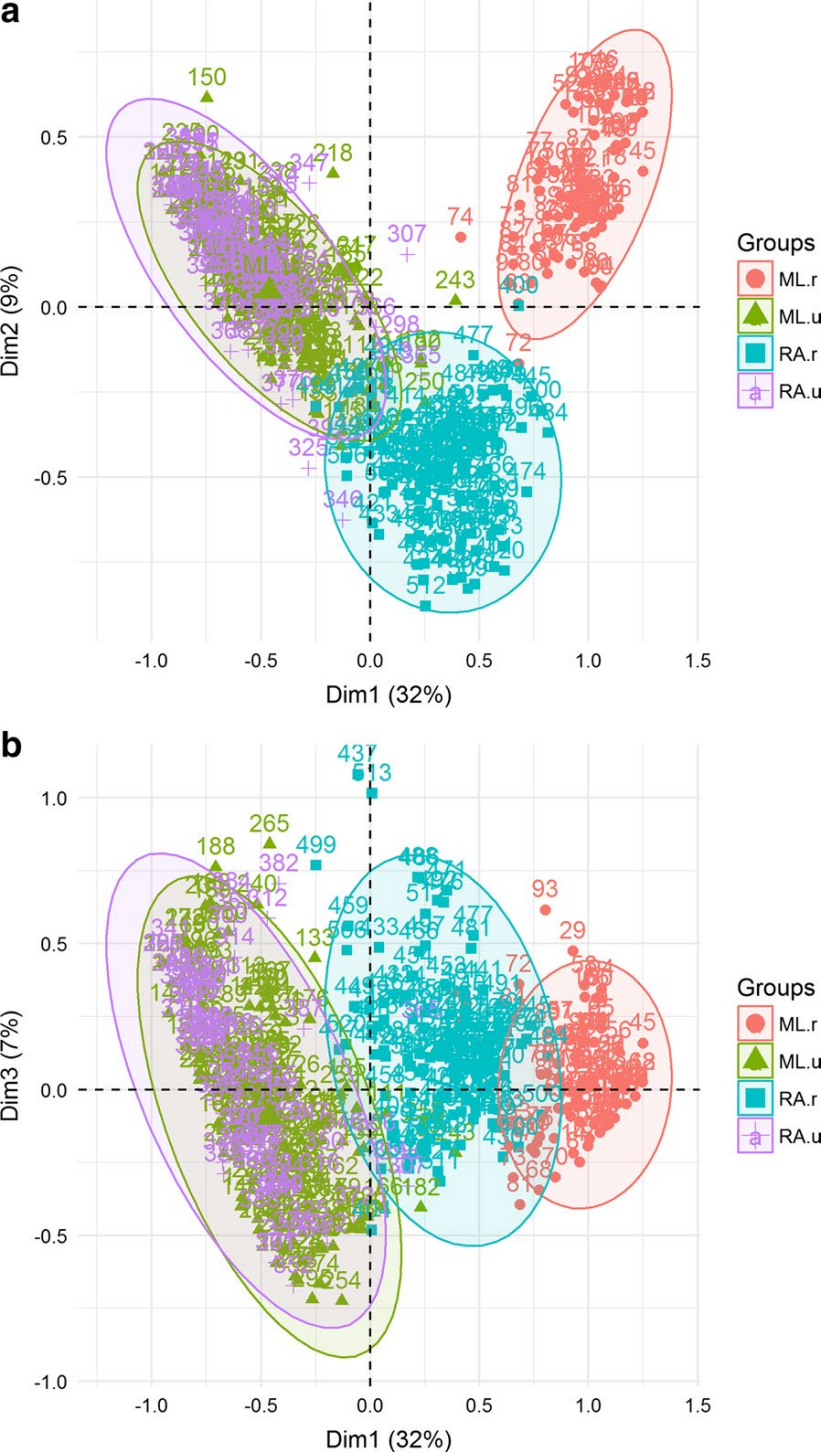
Distribution of households in **a** 1 × 2 dimension MCA factor map, **b** 1 × 3 dimension MCA factor map. Colors and circles refer to the location of the households in the four zones (ML-u and RA-u are the two urban areas, ML-r is the riverine area and RA-r is the road accessible rural area)

The first three MCA dimensions explained almost 50% of the variability among households. Dimension 1 contributed with 32% of the inertia, while dimension 2, contributed 9%, and dimension 3 with 7.04% (Fig. 3). These three dimensions were retained for the analysis. Figure 4a shows the V-shaped distribution of households along the first two MCA axes, with greater dispersion along the horizontal axis. In the MCA plot, the origin represents the average household and the dispersion around it indicates how they differ in relation to this average. Households from the two towns are clustered together in the second quadrant. The rural households aggregate in two clusters. One cluster, farthest to the right, is formed by households from the riverine zone (ML-r). The second cluster includes most households from the rural localities accessible by road (RA-r) and shows an overlap with the cluster formed by the two towns. Households at the intersection of these two clusters are located at Iracema (sigla.loc_IRA) and Pé-da-Terra (sigla.loc_PET), both at the periphery of ML. Figure 4b shows the distribution of households along the second and third axes. In this graph, an overlap between riverine and road accessible rural areas is observed. Variables defining the first dimension were associated with the availability of infrastructure and services, such as household accessibility via road or river, garbage disposal, source of water for domestic usage, and power grid electricity (Tables 2, 3; Additional file 3). Some variables directly associated with malaria exposure also contributed to the definition of this gradient, including the frequency at which householders entered the forest, worked in agriculture, had a boat, had crevices in their house walls. Income, measured in terms of ownership of non-essential appliances, such as washing machine, iron, blender, also contributed to this dimension. Overall, households at the high end of dimension 1 were accessible only by river, consumed water directly from the river, did not have bathrooms, had wood walls with crevices, electricity was often dependent on fuel-powered electric generator, relied on social benefits, and worked in family agriculture. In summary, they represent the households along rivers. Houses at the low end of dimension 1 were made of bricks without crevices, had piped water, bathroom with toilet, non-essential appliances, and received power grid electricity. They were accessed by paved roads, and had garbage collection. Most householders did not work in agriculture, nor required social benefits. From these features, dimension 1 can be interpreted as a gradient from the most developed areas and richer households found in the urban localities, to the most underdeveloped areas and poorer households located at the riverine localities (an urbanization/rurality gradient) (Additional file 3).

**Table 3:** Relative contributions of the variables to the 3 first dimensions of the MCA

Block	Categories	Dim 1		Dim 2		Dim 3	
		Contr.	R2	Contr.	R2	Contr.	R2
F	tv_NO	2.00	0.47	6.26	0.24	0.70	− 0.06
	tv_YES	0.25	− 0.47	0.80	− 0.24	0.09	0.06
	washing mach_NO	2.41	0.41	1.03	0.08	0.68	− 0.05
	washing mach_YES	1.70	− 0.41	0.72	− 0.08	0.48	0.05
	iron_NO	1.19	0.39	0.12	− 0.04	1.13	− 0.08
	iron_YES	2.31	− 0.39	0.23	0.04	2.19	0.08
	blender_NO	2.11	0.37	0.92	0.07	2.02	− 0.08
	blender_YES	1.12	− 0.37	0.48	− 0.07	1.07	0.08
	motor boat_NO	1.23	− 0.39	0.00	–	0.90	− 0.07
	motor boat_YES	2.24	0.39	0.01	–	1.65	0.07
	ox_NO	0.58	− 0.36	0.08	0.04	0.56	− 0.08
	ox_YES	1.82	0.36	0.28	− 0.04	1.78	0.08
	fishpond_NO	0.02	− 0.16	0.45	0.21	0.61	− 0.19
	fishpond_YES	0.21	0.16	4.39	− 0.21	5.92	0.19
G	agriculture_NO	1.260	− 0.41	0.04	–	0.40	− 0.05
	agriculture_YES	2.59	0.41	0.09	–	0.82	0.05
H	ceiling lining_NONE	0.38	0.39	0.11	− 0.08	3.75	− 0.27
	ceiling lining_PART	0.26	− 0.005	0.14	–	4.21	0.06
	ceiling lining_ALL	1.07	− 0.39	1.95	0.19	7.22	0.20
	wall_WOOD	0.49	0.41	0.58	− 0.13	2.10	− 0.19
	wall_BRICK	2.09	− 0.41	2.47	0.13	8.93	0.19
	cracks_NO	2.23	− 0.40	1.97	0.11	7.25	0.16
	cracks_YES	0.69	0.40	0.61	− 0.11	2.24	− 0.16
I	access_PAVED.ROAD	3.41	− 0.74	1.35	0.09	0.95	− 0.08
	access_RIVER	4.99	0.74	8.32	0.36	0.02	–
	access_DIRT.ROAD	0.35	− 0.003	16.9	− 0.44	1.46	0.08
	road.traffic.rain_NO	0.23	0.03	7.78	− 0.49	0.16	–
	road.traffic.rain_NO.HAVE	4.72	0.61	8.28	0.45	0.05	–
	road.traffic.rain_YES	1.81	− 0.64	0.49	0.05	0.06	–
JL	piped.water_INSIDE	1.43	− 0.41	0.02	–	3.93	0.15
	piped.water_OUTSIDE	0.10	− 0.14	2.21	− 0.15	7.73	− 0.21
	piped.water_NO.HAVE	2.74	0.56	1.65	0.13	0.29	0.06
	public.ser.water_NO	2.92	0.48	0.33	–	2.12	0.09
	public.ser.water_YES	2.92	− 0.48	0.33	0.05	2.12	− 0.09
	river.water_NO	1.64	− 0.53	0.20	–	0.35	− 0.05
	river.water_YES	4.17	0.53	0.52	–	0.89	0.05
	physio.needs_AREA	1.02	0.46	0.07	–	0.005	–
	physio.needs_SEPTIC	0.88	0.14	1.30	− 0.087	3.01	− 0.11
	physio.needs_TOILET	2.36	− 0.61	1.86	0.13	3.59	0.13
	place.wash.dishes_OUT	1.17	0.37	0.21	− 0.04	2.41	− 0.12
	place.wash.dishes_IN	2.05	− 0.37	0.36	0.04	4.22	0.12
	inap.garb.disposal_NO	3.41	− 0.50	1.51	0.09	1.16	− 0.06
	inap.garb.disposal_YES	2.85	0.50	1.27	− 0.09	0.97	0.06
	garb.collect_NO	3.95	0.55	1.31	− 0.09	0.82	0.06
	garb.collect_YES	3.63	− 0.55	1.20	0.09	0.75	− 0.06
M	electricity _GENERATOR	2.93	0.35	2.89	0.02	0.22	–
	electricity _NET	1.27	− 0.81	2.24	− 0.33	0.01	–
	electricity_NO	1.876	0.46	6.89	0.32	0.08	–
NO	displac.benefits_NO	2.56	− 0.45	1.22	0.09	1.57	− 0.08
	displac.benefits_YES	2.60	0.45	1.24	− 0.09	1.59	0.08
	freq.into.forest_SOMETIMES	0.07	− 0.13	2.89	− 0.17	0.10	–
	freq.into.forest_NEVER	2.05	− 0.48	0.75	0.09	1.39	− 0.09
	freq.into.forest_ALWAYS	3.41	0.61	0.36	0.08	1.02	0.07

Households did not spread as much along the second dimension as they did along the first one, differed mainly in their access, source of energy, and working activity. At the high end of the second axis, households were only accessible by river and were not power-grid-energy served (Additional file 3). At the low end of the second axis are rural households accessible by roads, with access to electricity and people working on aquaculture and agriculture activities. This second dimension discriminated between the riverine and road accessible rural households (Table 3; Additional file 3). While the riverine population works most in agriculture, the road rural population works in fish farming (a gradient of rural differentiation or riverine-road rural gradient).

The third dimension explained 7.04% of the variance. Despite the low variance, this dimension is important to discriminate households within the urban and road-accessible rural localities. The variables that most contributed to this axis are related to the physical characteristics of the house (materials) as well as their source of water. At the positive side of this axis, there are households made of brick, fully covered with roof lining, no crevices in their walls, piped water, bathroom with toilet, and fish ponds. At the negative side of the 3rd axis, households are made of wood with crevices, no roof lining, no bathroom, piped water only outside the house, no fishponds. From these features, the 3rd axis is interpreted as a housing construction gradient, ranging from primitive wood houses without plumbing to fully equipped brick houses. This gradient exists both in the towns and the road accessible zones, but not in the riverine zone (Fig. 4b).

### Malaria distribution along the development gradients

**Fig. 5:**
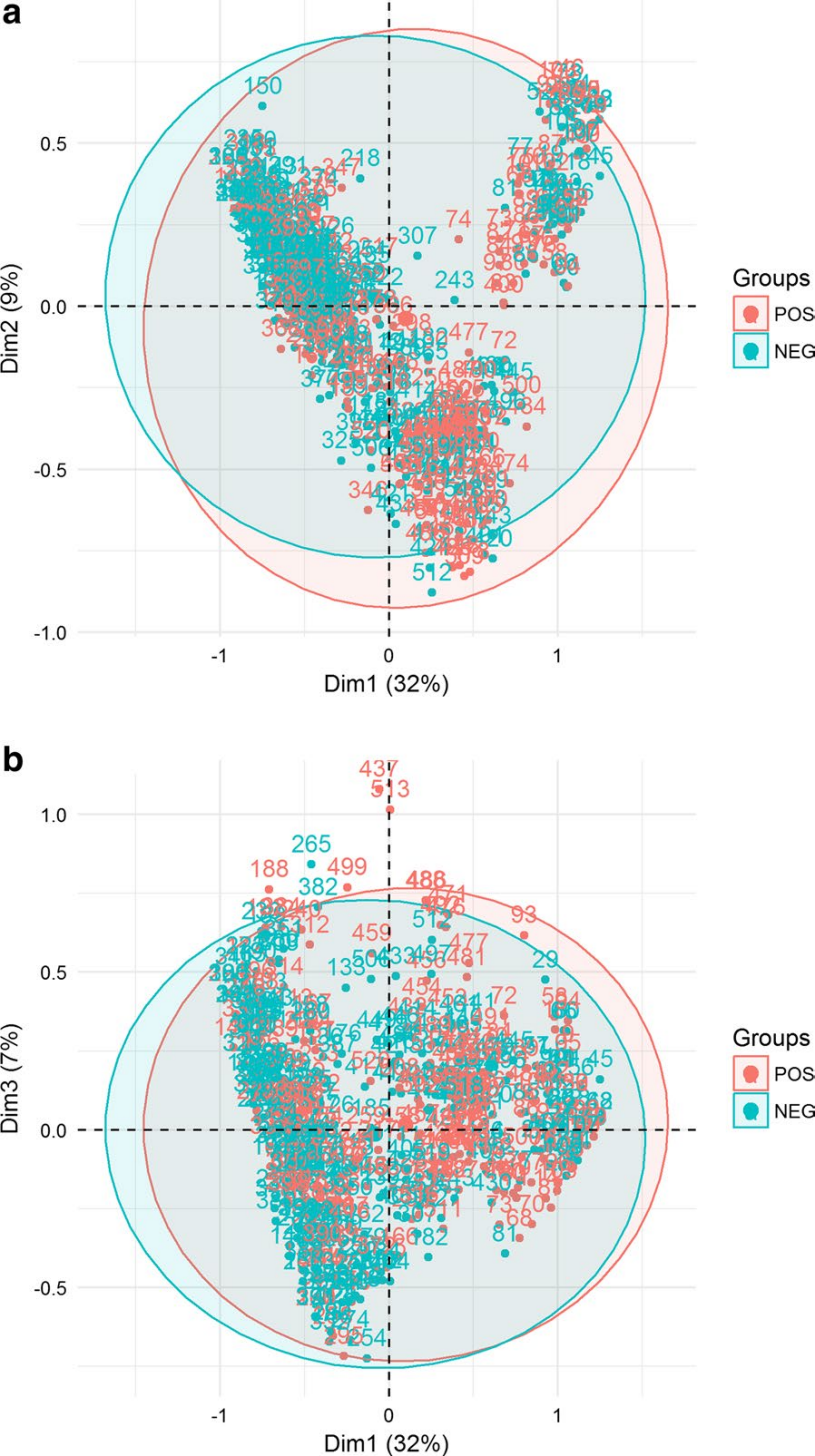
Distribution of households in the **a** 1 × 2 dimension MCA factor map, and **b** the 1 × 3 dimension MCA factor map. Colors indicate the status of the household according to the presence of at least one member with malaria in the last 12 months

**Fig. 6:**
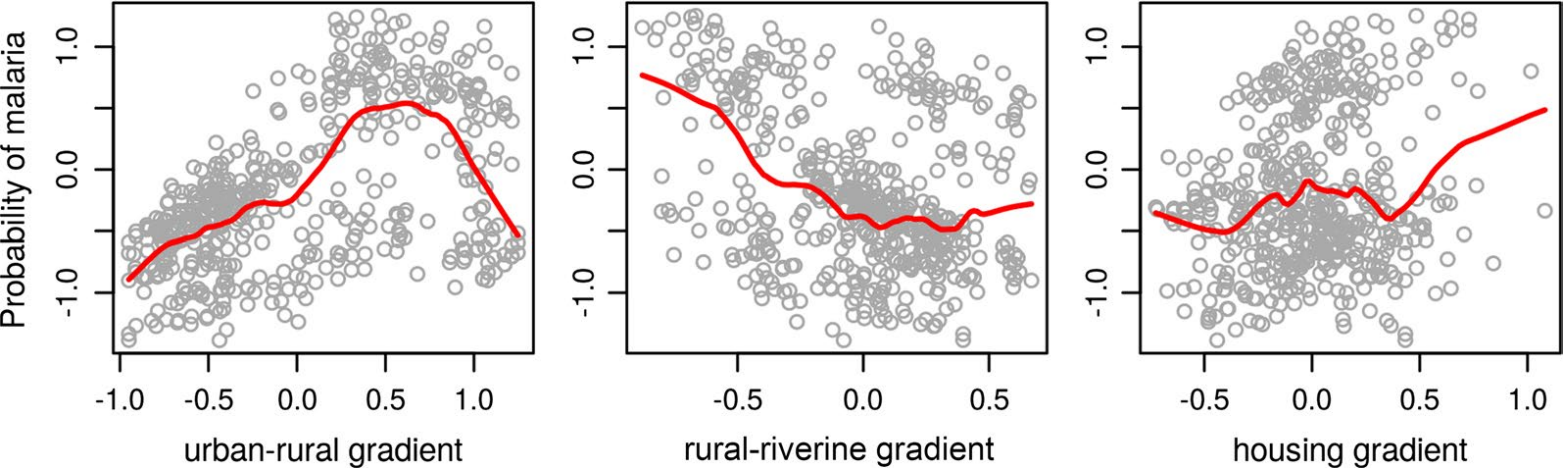
Predicted probability of malaria along the three development gradients produced by the multivariate analysis

Figure 5 shows the distribution of households with and without *malaria in the last 12 months* in the 1 × 2 and 1 × 3 MCA factor maps. Although malaria does not cluster on any specific region, there is a slight trend towards the fourth quadrant of the 1 × 2 factor map (Fig. 5a) and the fourth and first quadrants of the 1 × 3 factor map (Fig. 5b). According to the mixed logistic regression model, the odds of observing a household with malaria increased significantly along the first MCA dimension (Table 4). The marginal probability of malaria along this axis ranged from ca. 30% in the most developed urban household to ca. 65% in the most forested primitive household (Additional file 4). Along the second dimension, there was a weak negative effect (p = 0.10), with the odds of having malaria increasing in households at the road accessible localities. Variation in malaria probability was lower in comparison with the first gradient. The third MCA dimension was not significantly associated with malaria. Figure 6 shows the predicted probability of malaria along the three MCA gradients.

**Table 4:** Odds ratio of having a household with malaria cases along the three development gradients derived from the MCA

Variable	Odds ratio	95% CI OR	p value
Urban–rural gradient (MCA Dim1)	1.92	(1.23–3.02)	0.004
Rural-riverine gradient (MCA Dim 2)	0.55	(0.26–1.12)	0.10
Intra urban (un)development gradient (MCA Dim 3)	1.46	(0.75–2.82)	0.25

## Discussion

This study describes the micro-epidemiology of malaria within one of the most important malaria endemicity pockets in the Americas, where 3 municipalities contributed to the top 20 cities with the highest rates of malaria between 2012 and 2014 [2, 10, 23]: Mâncio Lima with 341.9 Annual Parasitic Index (API), Rodrigues Alves with 263.6 API and Cruzeiro do Sul with 195.2 API, in 2014 [23].

Three dimensions of development emerged from the household data, characterizing large independent axes of variation in living conditions in the region. The first dimension describes an index of rurality, with households at the low end of the scale is relatively urban, and the ones at the high end, the most rural. A continuous gradient of rurality has advantages over the traditional urban–rural dichotomy avoiding ’threshold traps’ where households at the border are at risk of misclassification. As an objective concept, it can be used to trek the trajectories of rurality change over time [24]. At the locality level, greater rurality was associated with lower population size, lower population density, lower extent of built-in area and greater remoteness in relation to administrative centers, agreeing with the main dimensions considered by Waldorf [24].

The second dimension found in the study describes the different household conditions in riverine and road-accessible rural localities. It emphasizes differences in productive assets held by the household (having a fish pond), as well as communal assets to which they have access (roads and power grid electricity) that facilitate access to industrialized materials for house construction. In poor rural communities, these assets make their physical capital, which together with the social, human, natural and financial capitals, compose the household wealth [25]. Households along the rivers Moa and Azul had the lowest physical capital, in comparison to those accessible by roads. Lack of physical capital can be partially explained by the landscape. First, the transportation of goods in small boats along these rivers is costly and difficult. Second, available industrialized materials for house construction are not suitable for the flooded forest. These regions require specific malaria-proof housing solutions.

The third axis of development is an index of housing construction quality. Variation in this index is observed within the road accessible localities, but not within the river accessible households, due to the constraints above described. Housing construction quality is a proxy for wealth in several studies of malaria risk factors, as reviewed by Bannister-Tyrrell et al. [14].

All three development dimensions were required for the malaria probability model. The probability of malaria ranged from a minimum of 30% in the least rural (more urban) households, peaked at the households with intermediate rurality (60%) and decreased to ca. 50% in the most rural households. There is no evidence of a sharp transition in malaria risk between urban and rural localities that would suggest distinct levels of exposure. A stable spatial gradient encompassing all levels of rurality is of interest for management. This spatial pattern is consistent with malaria maintained in a source-sink metapopulation structure where localities with high and low transmission conditions are connected by human commutation [26]. The role of each locality as source or sink is not clear, however. Despite the low probability of malaria in the urban localities, it is known that localities in ML-u can maintain a large population of *Anopheles darlingi* and *Anopheles albitarsis* by their fish farming activities [12]. If this is sufficient to characterize the urban localities as sources, it is not known. The households with the highest probability of malaria were located at the end of the secondary roads, close to the deforestation borders. A similar pattern was observed by Silva-Nunes et al. [27] in the Acrelândia (Acre state) malaria hotspot, where a higher risk of malaria was associated with new plots in the periphery of more structured areas, generally occupied by new settlers from non-malaria regions. This is a pattern that seems to persist in the Amazon Basin and call for rethinking the current national colonization programme [28]. These populations are not isolated, but linked to each other by mobility, which enhances the importation of *Plasmodium* between hotspots and other regions [29], especially in regions of unstable populations that commute [10] in search of basic services such as health, banking and education, or for work [9] as the case of ML urban zone, the main attraction place of the study area. Fish farming is also found throughout the localities at intermediate rurality. This activity can contribute to maintaining transmission away from the forest frontier. Critical community size (CCS) is another factor that affects the stability of malaria. Localities with very small household density, as those found in the riverine region, may have environmental conditions for transmission, but not the minimum amount of individuals to sustain it. However, accurate information about CCS is somewhat challenging to achieve, as it is influenced by immunity, migrations, and other variables [30].

Characterizing the microgeography of the Juruá malaria hotspot is essential for better understanding the factors that maintain transmission and to design adequate control strategies. The place where one lives is a risk factor for malaria [27, 31]. In this study, it was detected the risk of malaria is higher for poorer individuals living in rural areas than for poorer individuals living in urban areas. Overall, the region presented low rates of development and poor infrastructure indicators such as water supply and adequate garbage disposal [15]. The control of malaria and other diseases is further challenged by the fact that most people live within or very close to environmentally protected areas and require specifically tailored strategies.

The study relied on self-reported malaria and third-party information. This may be a source of bias. However, the population is familiar with malaria symptoms which are debilitating enough to be remembered. Another potential source of bias is related to the sampling scheme. Designing a sampling strategy for this heterogeneous population presented several challenges, as discussed in Kondo et al. [32]. The population is highly mobile and scattered; available population counts were imprecise. Whole localities disappear while new ones are created in a few years, householders spend months away from their primary address either working or studying or can move their wood houses from one place to the other, as necessary. Despite these limitations, this study has provided demographic results that can inform future, more detailed, complex and expensive studies using serological techniques and genetic markers.

## Conclusion

This work contributed to the characterization of one of the most important endemicity pockets in the Americas and provides a wealth of information that can effectively be used for the overall goal of malaria elimination. The methodological approach using MCA proved useful for mapping the distribution of living conditions associated with malaria. There is no sharp transition in the risk of malaria between rural and urban localities, and interventions should not deal separately with the urban and rural zones. Finally, the characterization of this population is of great relevance for Brazil, since it is a hard to reach population, living in extreme poverty in a situation of invisibility. Understanding local habits, identifying populations at greater social vulnerability is essential for combating poverty and improving the living conditions of this population.

## Additional files

**Additional file 1.** Information of the study localities.

**Additional file 2.** Images of exemplification of the types of localities in the study area.

**Additional file 3.** Complementary MCA plots.

**Additional file 4.** Details of mixed logistic regression models analyzes.

## References

[1] WHO. World Malaria Report 2013. Technical report. Geneva: World Health Organization; 2013. http://www.who.int/malaria/publications/world_malaria_report_2013/en/.

[2] Ferreira MU, Castro MC (2016). Challenges for malaria elimination in Brazil. Malar J..

[3] Rosas-Aguirre A, Gamboa D, Manrique P, Conn JE, Moreno M, Lescano AG (2016). Epidemiology of *Plasmodium vivax* malaria in Peru. Am J Trop Med Hyg..

[4] Brien ATO, Ramírez JF, Martínez SP (2014). A descriptive study of 16 severe *Plasmodium vivax* cases from three municipalities of Colombia between 2009 and 2013. Malar J..

[5] Chu CS, White NJ (2016). Management of relapsing *Plasmodium vivax* malaria. Expert Rev AntiInfect Ther..

[6] Vitor-Silva S, Siqueira AM, Sampaio VS, Guinovart C, Reyes-Lecca RC, Melo GC (2016). Declining malaria transmission in rural Amazon: changing epidemiology and challenges to achieve elimination. Malar J..

[7] Oliveira-Ferreira J, Lacerda MVG, Brasil PP, Ladislau JJLB, Tauil PL, Daniel-Ribeiro CCT (2010). Malaria in Brazil: an overview review. Malar J..

[8] da Silva-Nunes M, Moreno M, Conn JE, Gamboa D, Abeles S, Vinetz JM, Ferreira MU (2012). Amazonian malaria: asymptomatic human reservoirs, diagnostic challenges, environmentally driven changes in mosquito vector populations, and the mandate for sustainable control strategies. Acta Trop..

[9] Parise EV, de Araújo GC, Pinheiro RT (2011). Análise espacial e determinação de áreas prioritárias para o controle da malária, no Estado no Tocantins, 2003–2008. Rev Soc Brasil Med Trop..

[10] Valle D, Tucker Lima JM (2014). Large-scale drivers of malaria and priority areas for prevention and control in the Brazilian Amazon region using a novel multi-pathogen geospatial model. Malar J..

[11] Olson SH, Gangnon R, Silveira GA, Patz JA (2010). Deforestation and malaria in Mâncio Lima County Brazil. Emerg Infect Dis..

[12] Reis IC, Codeço CT, Degener CM, Keppeler EC, Muniz MM, Oliveira FGS (2015). Contribution of fish farming ponds to the production of immature *Anopheles* spp. in a malaria-endemic Amazonian town. Malar J..

[13] Wilson ML, Krogstad DJ, Arinaitwe E, Arevalo-Herrera M, Chery L, Ferreira MU (2015). Urban malaria: understanding its epidemiology, ecology, and transmission across seven diverse ICEMR network sites. Am J Trop Med Hyg.

[14] Bannister-Tyrrell M, Verdonck K, Hausmann-Muela S, Gryseels C, Ribera JM, Grietens KP (2017). Defining micro-epidemiology for malaria elimination: systematic review and meta-analysis. Malar J..

[15] IBGE: Instituto Brasileiro de Geografia e Estatística- Cidades; 2010. http://www.cidades.ibge.gov.br. Accessed 15 July 2014.

[16] Martins AC, Araújo FM, Braga CB, Guimarães MGS, Nogueira R, Arruda RA (2015). Clustering symptoms of non-severe malaria in semi-immune Amazonian patients. Peer J..

[17] Greenacre M, Blasius J (2006). Multiple correspondence analysis and related methods.

[18] Ayele D, Zewotir T, Mwambi H (2014). Multiple correspondence analysis as a tool for analysis of large health surveys in African settings. Afr Health Sci..

[19] Sourial N, Wolfson C, Zhu B, Quail J, Fletcher J, Karunananthan S (2010). Correspondence analysis is a useful tool to uncover the relationships among categorical variables. J Clin Epidemiol.

[20] Lê S, Josse J, Husson F (2008). FactoMineR: an R package for multivariate analysis. J Stat Softw.

[21] R Core Team. R: a language and environment for statistical computing. Vienna: R Foundation for Statistical Computing; 2017. https://www.R-project.org/.

[22] Bates D, Maechler M, Bolker B, Walker S, Christensen RHR, Singmann H (2014). Linear mixed-effects models using ’Eigen’ and S4. LME4.

[23] PAHO. Report on the situation of malaria in the Americas 2014. Technical report. Washington, DC: Pan American Health Organization; 2016. http://www.romeurope.org/IMG/Rapport en anglais- sans commentaire.pdf.

[24] Waldorf BS. A continuous multi-dimensional measure of rurality: moving beyong the threshold measures. In: Annual Meeting, Long Island, California; 2006. p. 1–29.

[25] de Sherbinin A, VanWey LK, McSweeney K, Aggarwal R, Barbieri A, Henry S (2008). Rural household demographics, livelihoods and the environment. Glob Environ Change..

[26] Rascalou G, Pontier D, Menu F, Gourbière S (2012). Emergence and prevalence of human vector-borne diseases in sink vector populations. PLoS ONE..

[27] Silva-Nunes M, Codeço CT, Malafronte RS, Da Silva NS, Juncansen C, Muniz PT (2008). Malaria on the Amazonian frontier: transmission dynamics, risk factors, spatial distribution, and prospects for control. Am J Trop Med Hyg..

[28] Sampaio VS, Siqueira AM, Alecrim MdGC, Mourão MPG, Marchesini PB, Albuquerque BC (2015). Malaria in the state of Amazonas: a typical Brazilian tropical disease influenced by waves of economic development. Rev Soc Brasil Med Trop..

[29] Wesolowski A, Eagle N, Tatem AJ, Smith DL, Noor AM, Snow RW (2012). Quantifying the impact of human mobility on malaria. Science..

[30] Chowell G, Munayco CV, Escalante AA, McKenzie FE (2009). The spatial and temporal patterns of falciparum and vivax malaria in Perú: 1994–2006. Malar J..

[31] Chuquiyauri R, Paredes M, Peñataro P, Torres S, Marin S, Tenorio A (2012). Socio-demographics and the development of malaria elimination strategies in the low transmission setting. Acta Trop..

[32] Kondo MC, Bream KDW, Barg FK, Branas CC (2014). A random spatial sampling method in a rural developing nation. BMC Public Health.

