# Correction to: Socioeconomic and demographic characterization of an endemic malaria region in Brazil by multiple correspondence analysis

**DOI:** 10.1186/s12936-017-2058-7

**Published:** 2017-10-11

**Authors:** Raquel M. Lana, Thais I. S. Riback, Tiago F. M. Lima, Mônica da Silva Nunes, Oswaldo G. Cruz, Francisco G. S. Oliveira, Gilberto G. Moresco, Nildimar A. Honório, Cláudia T. Codeço

**Affiliations:** 1 Programa de Pós-Graduação em Epidemilogia em Saúde Pública, Escola Nacional de Saúde Pública Sérgio Arouca, Fundação Oswaldo Cruz, Rua Leopoldo Bulhões, 1480, Manguinhos, Rio de Janeiro, RJ 21041-210 Brazil; 2 Programa de Computação Científica, Fundação Oswaldo Cruz, Residência Oficial, Avenida Brasil, 4365, Manguinhos, Rio de Janeiro, RJ 21040-360 Brazil; 3 Laboratório de Engenharia e Desenvolvimento de Sistemas, Departamento de Computação e Sistemas, Instituto de Ciências Exatas e Aplicadas, Universidade Federal de Ouro Preto., Rua 36, n. 115, Loanda, João Monlevade, MG 35931-008 Brazil; 4 Centro de Ciências da Saúde, Universidade Federal do Acre, Campus Universitário-BR 364, km 4-Distrito Industrial, Rio Branco, AC 69920-900 Brazil; 5 Campus Cruzeiro do Sul, Universidade Federal do Acre, Estrada do Canela Fina, s/n, Cruzeiro do Sul, AC 69980-000 Brazil; 6 Coordenação Geral dos Programas Nacionais de Controle e Prevenção da Malária e das Doenças transmitidas pelo Aedes, Departamento de Vigilância das Doenças Transmissíveis, Secretaria de Vigilância em Saúde-Ministério da Saúde, SRTV 702, Via W 5 Norte, Ed. PO700-6 andar, Brasília, DF 70723-040 Brazil; 7 Laboratório de Mosquitos Transmissores de Hematozoários‑Lathema, Instituto Oswaldo Cruz, FIOCRUZ, Avenida Brasil, 4365, Manguinhos, Rio de Janeiro, RJ 21040-360 Brazil; 8 Núcleo Operacional Sentinela de Mosquitos Vetores-Nosmove/FIOCRUZ, Avenida Brasil, 4365, Manguinhos, Rio de Janeiro, RJ 21040-360 Brazil

## Correction to: Malar J (2017) 16:397 DOI 10.1186/s12936-017-2045-z

After publication of the article [1], it has been brought to our attention that the y-axis of Fig. 6 has been labeled incorrectly. It should read “linear predictor”. This has now been corrected in the original article.
