# Supplementary material for: Socioeconomic and demographic characterization of an endemic malaria region in Brazil by multiple correspondence analysis
Source: Malar J. 2017 Oct 2;16:397. doi: 10.1186/s12936-017-2045-z (PMC5625626; doi:10.1186/s12936-017-2045-z)
Supplement: Supplementary file 1 — Additional file 1. Information of the study localities. [file 12936_2017_2045_MOESM1_ESM.pdf]

**Additional file 1 - Information of the study localities.**

Description: Zone (r = rural, u = urban, ML = Mâncio Lima, RA = Rodrigues Alves), name and abbreviation.

| Zone | Localities' names | Abbreviation |
|---|---|---|
| ML.r | TRÊS UNIDOS | TRU |
| ML.r | NOVA LIÇÃO | NOL |
| ML.r | BOM SOSSEGO | BOS |
| ML.r | BARRO VERMELHO | BAV |
| ML.r | RIO AZUL-PDSSS | RIA |
| ML.r | SERRA DO MOA | SDM |
| ML.r | CONCEIÇÃO-PDSSS | CON |
| ML.r | SEDE-PDSSS | SED |
| ML.r | PROSPERIDADE-PDSSS | PROS |
| ML.r | ZUMIRA | ZUM |
| ML.r | SÃO FRANCISCO-PDSSS | SFP |
| ML.r | BOA VISTA-PDSSS | BOV |
| ML.r | SOCÓ | SOC |
| ML.r | PÉ-DA-TERRA | PET |
| ML.u | SÃO FRANCISCO | SFO |
| ML.u | BANDEIRANTE | BAN |
| ML.u | IRACEMA | IRA |
| ML.u | CENTRO | CEN |
| ML.u | JOSÉ MARTINS | JOM |
| ML.u | SÃO VIDAL | SAV |
| ML.u | BETÃNIA | BET |
| ML.u | GUARANI | GUA |
| RA.u | BAIRRO MANOEL GOMES | BMG |
| RA.u | BAIRRO CENTRO | BAC |
| RA.u | BAIRRO ROBERTO LEITE | BRL |
| RA.u | BAIRRO DARIO PEREIRA | BDP |
| RA.u | BAIRRO SÃO FRANCISCO | BSF |
| RA.r | PARANÁ DOS MOURAS | PDM |
| RA.r | PUCALPA | PUC |
| RA.r | LUZEIRO | LUZ |
| RA.r | VALQUÍRIA | VAL |
| RA.r | SERINGAL AGROVILA | SEA |
| RA.r | 13 DE MAIO | 1DM |
| RA.r | GLEBA PARANÁ | GLP |
| RA.r | RAMAL DO BACURI | RDB |
| RA.r | TORRE DA LUA | TDL |
| RA.r | SÃO PAULO | SAP |
| RA.r | SÃO FRANCISCO RAM | SFR |
| RA.r | NOVA CINTRA | NOC |
| RA.r | PROFETA | PROF |
