# Supplementary material for: Socioeconomic and demographic characterization of an endemic malaria region in Brazil by multiple correspondence analysis
Source: Malar J. 2017 Oct 2;16:397. doi: 10.1186/s12936-017-2045-z (PMC5625626; doi:10.1186/s12936-017-2045-z)
Supplement: Supplementary file 2 — Additional file 2. Images of exemplification of the types of localities in the study area. [file 12936_2017_2045_MOESM2_ESM.pdf]

**Additional file 2 - Images of exemplification of the types of localities in the study area.**

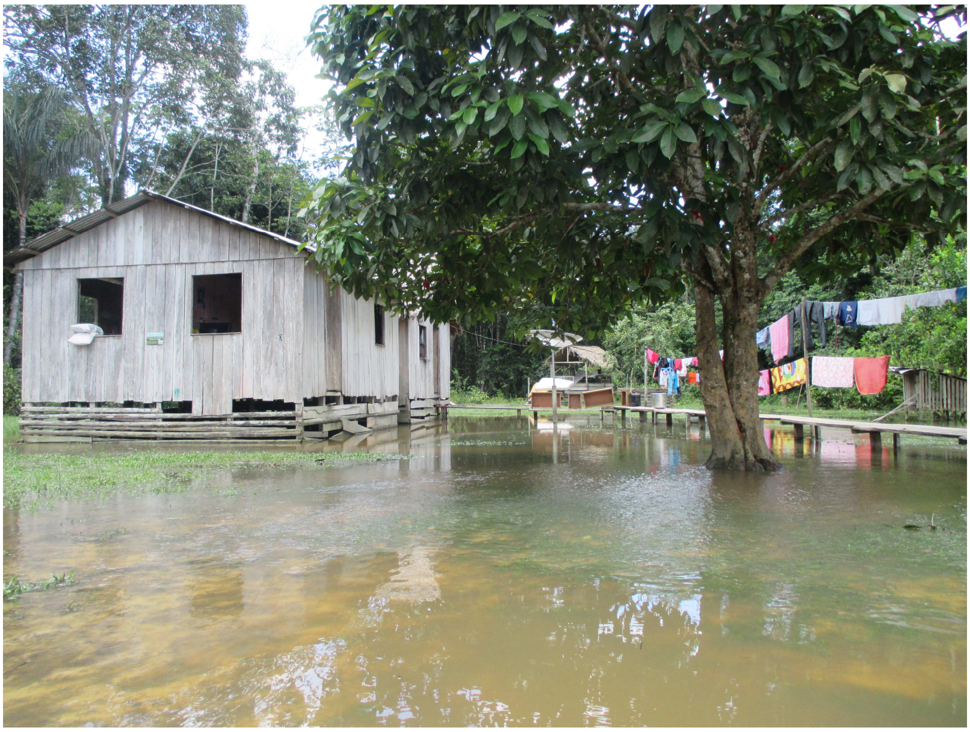

**Figure A21. A typical household for riverine population in ML.r.**

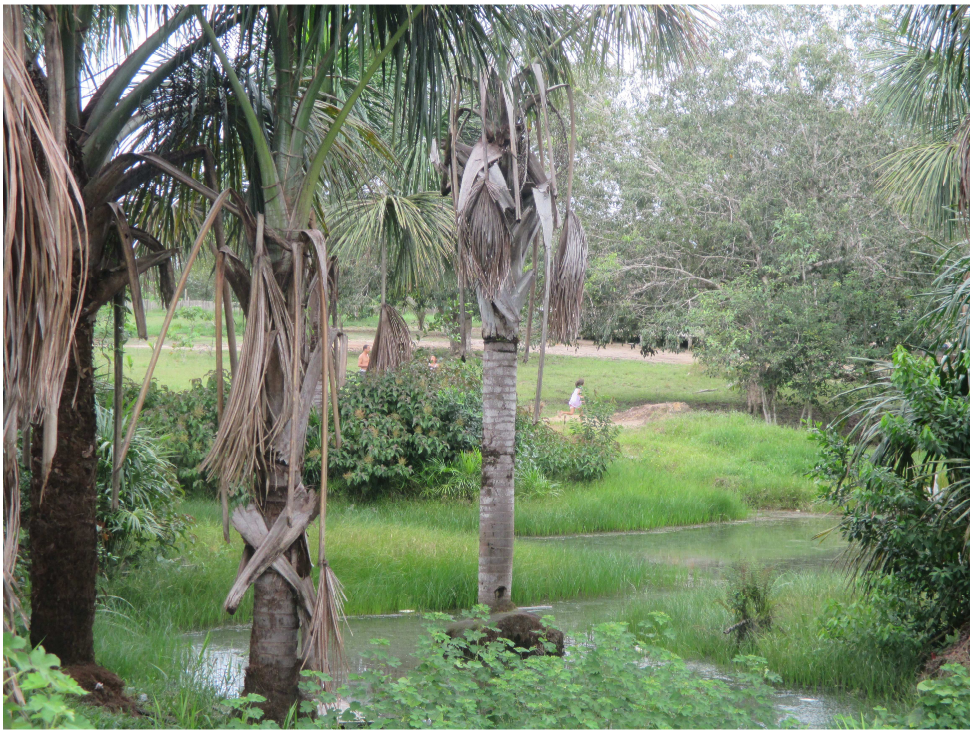

**Figure A22. A typical swamp in urban zones.**

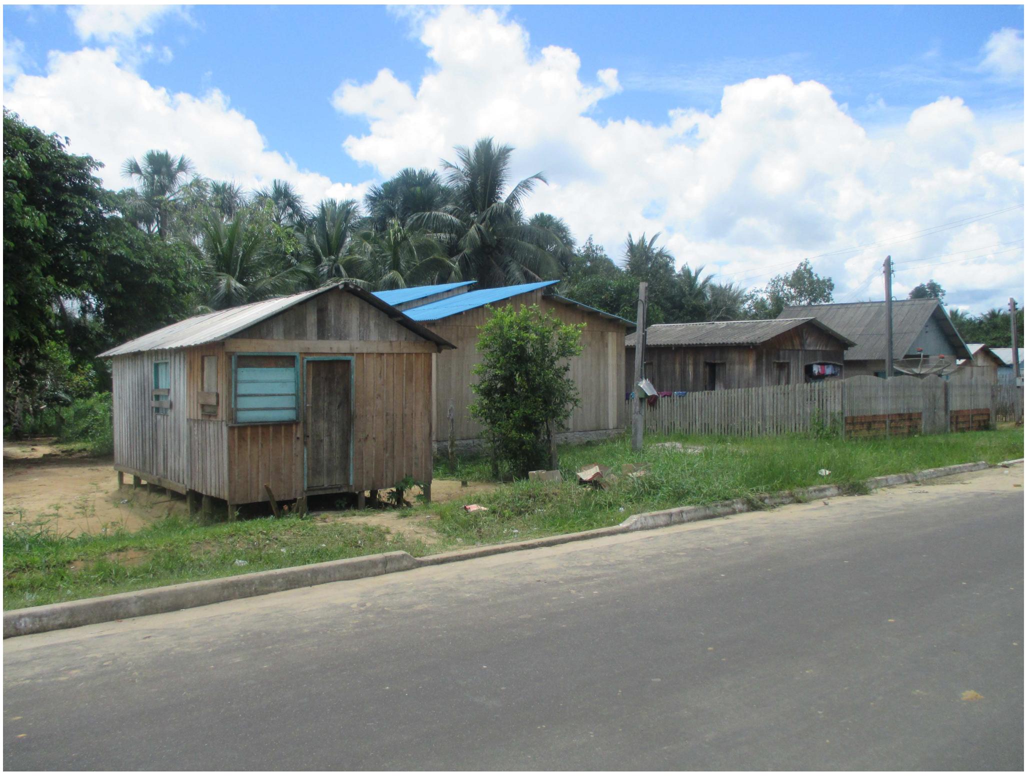

**Figure A23. Wood household in urban zones.**

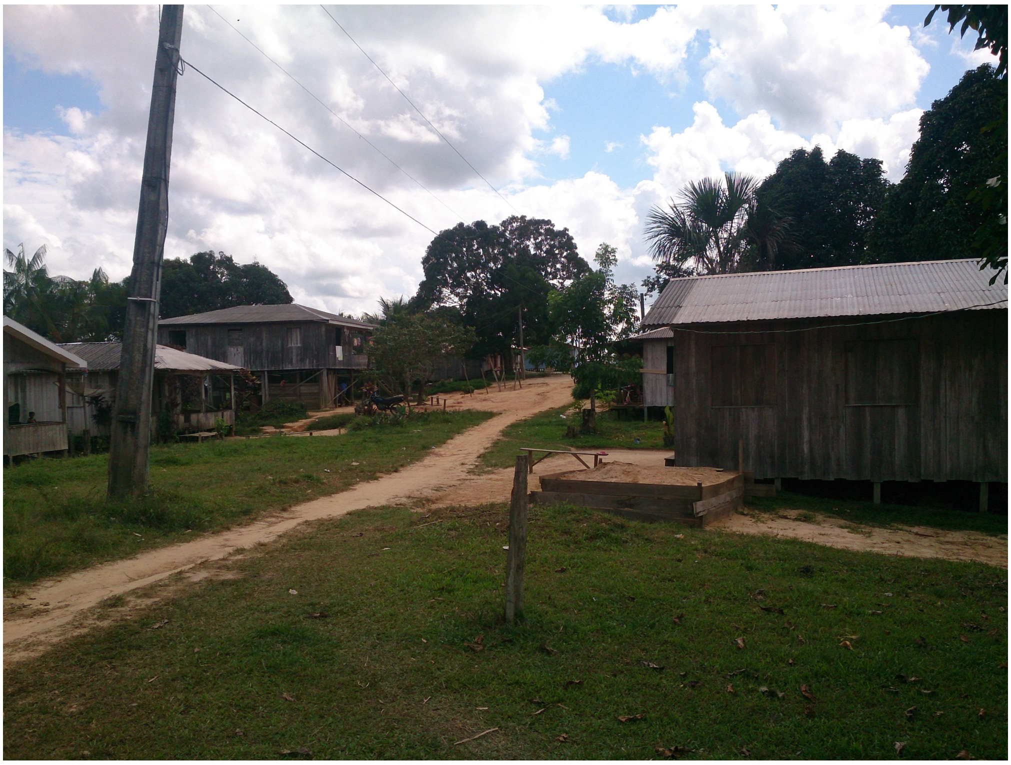

**Figure A24. Rural locality in RA.r.**

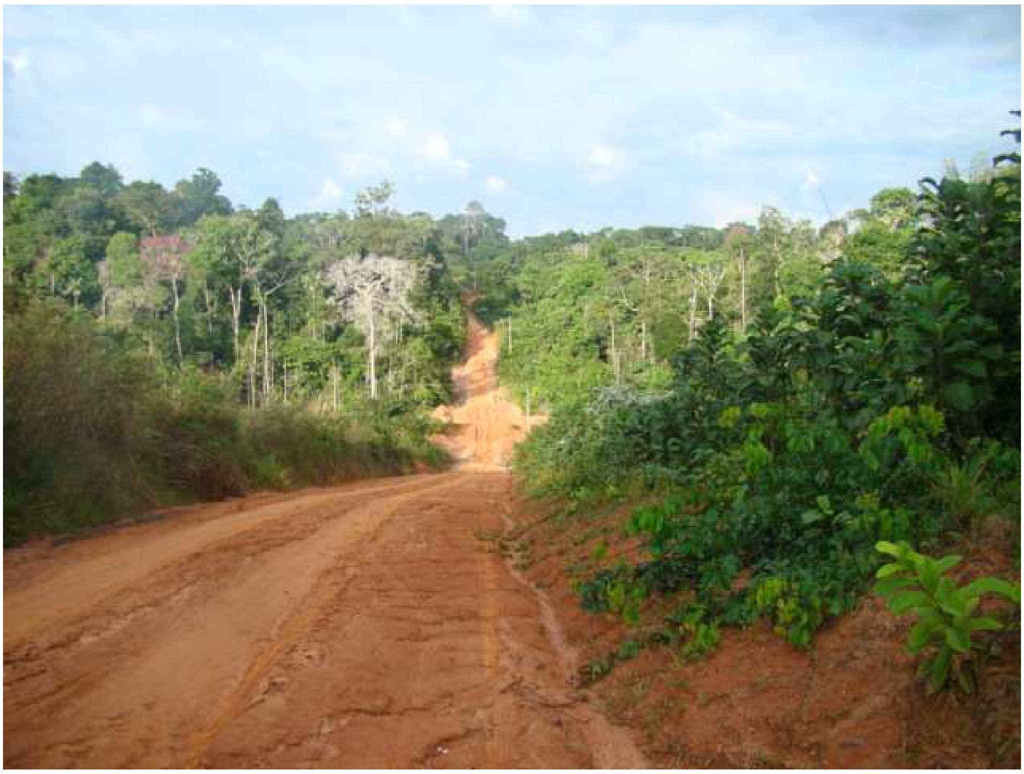

**Figure A25.** ***Ramais*** **(dirt road) in RA.r**
