# Supplementary material for: Socioeconomic and demographic characterization of an endemic malaria region in Brazil by multiple correspondence analysis
Source: Malar J. 2017 Oct 2;16:397. doi: 10.1186/s12936-017-2045-z (PMC5625626; doi:10.1186/s12936-017-2045-z)
Supplement: Supplementary file 3 — Additional file 3. Complementary MCA plots. [file 12936_2017_2045_MOESM3_ESM.pdf]

Additional file 3 - Complementary MCA plots.

Description: This file shows complementary plots for the MCA. Graphs 1 and 2 represent the variables and categories on factor map in final model. Graphs 3 and 4 represent the quality of representation of each household on the factor map of each household to the MCA map. Graphs 5 and 6 represent the supplementary variables on factor map. Values close to 1 represent good representation.

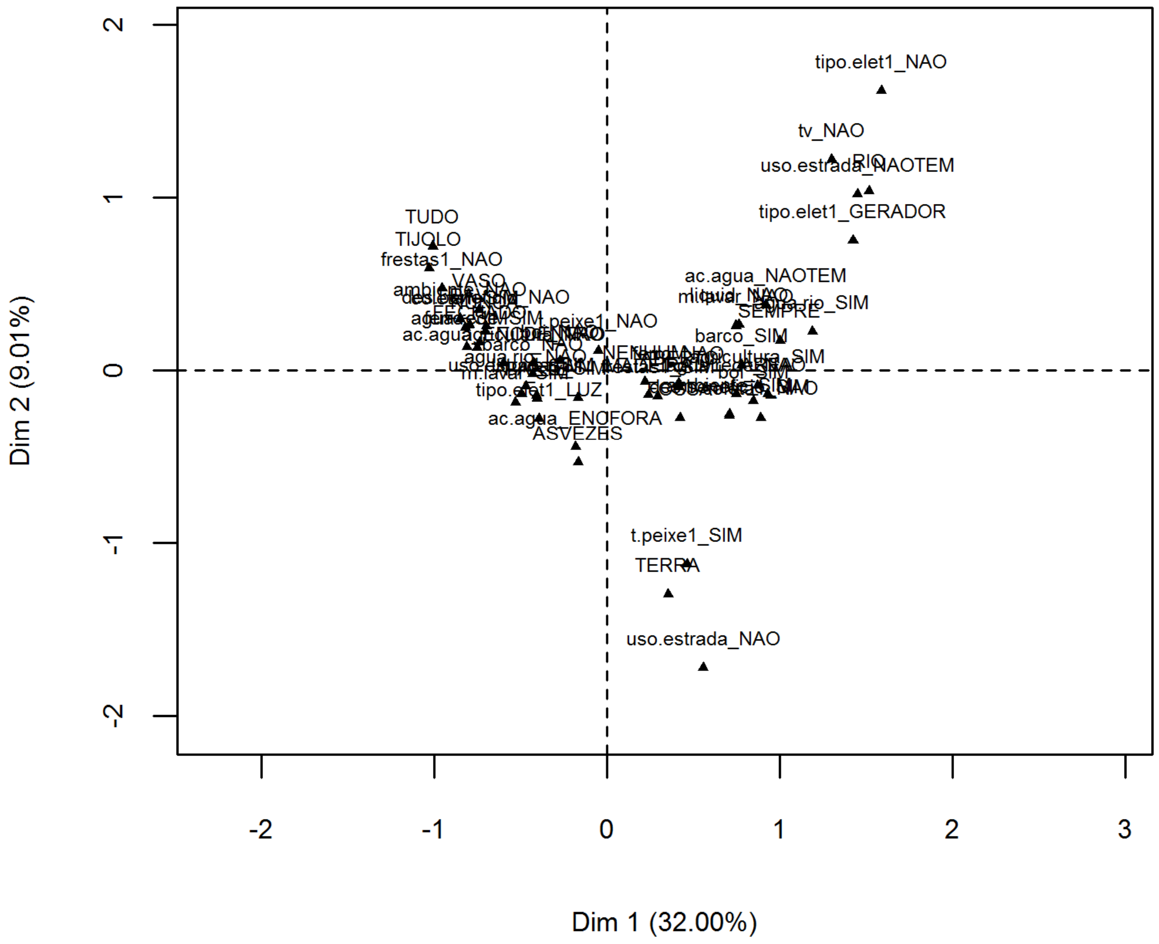

Figure A31. MCA plot showing the joint distribution of the categories in dimensions 1x2. See Table 2 for information on variable names.

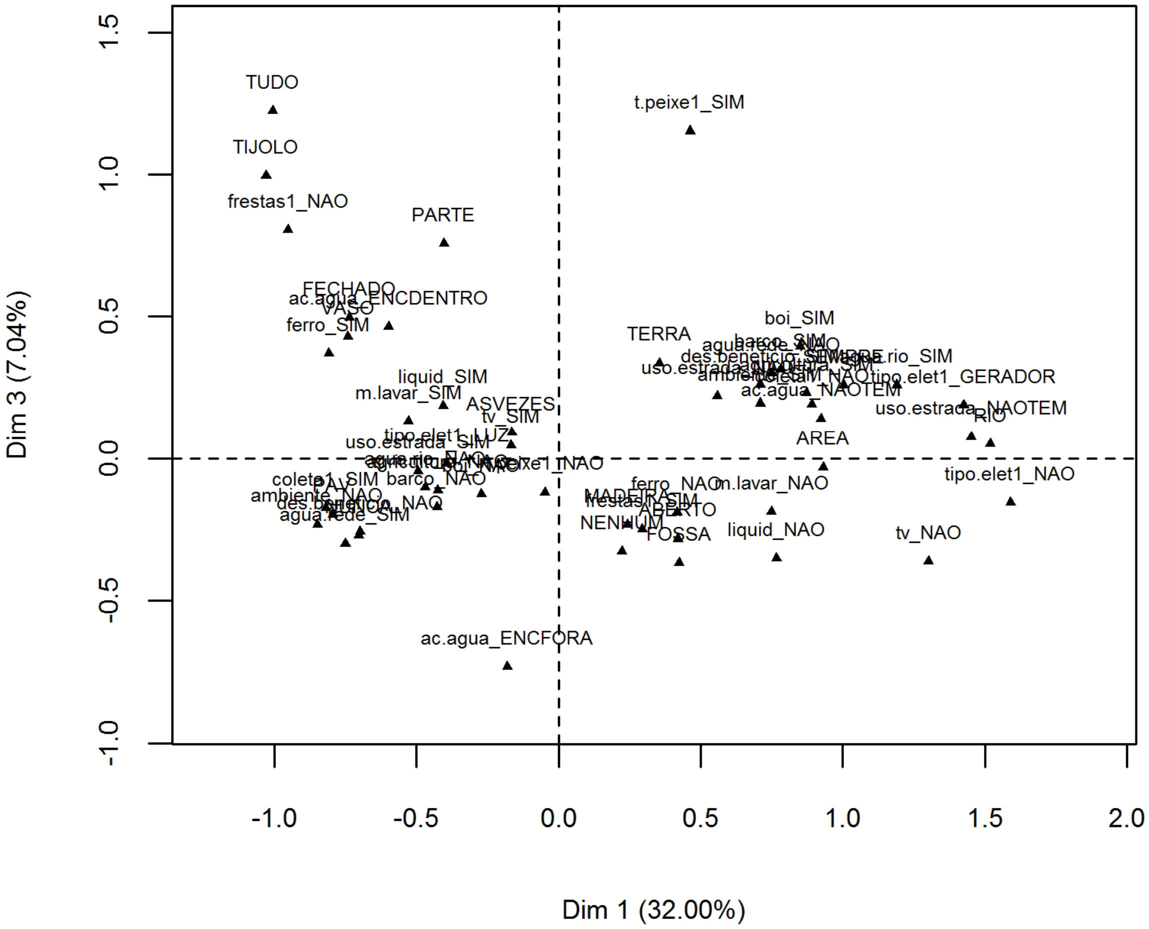

Figure A32. MCA plot showing the joint distribution of the categories in dimensions 1x3. See Table 2 for information on variable names.

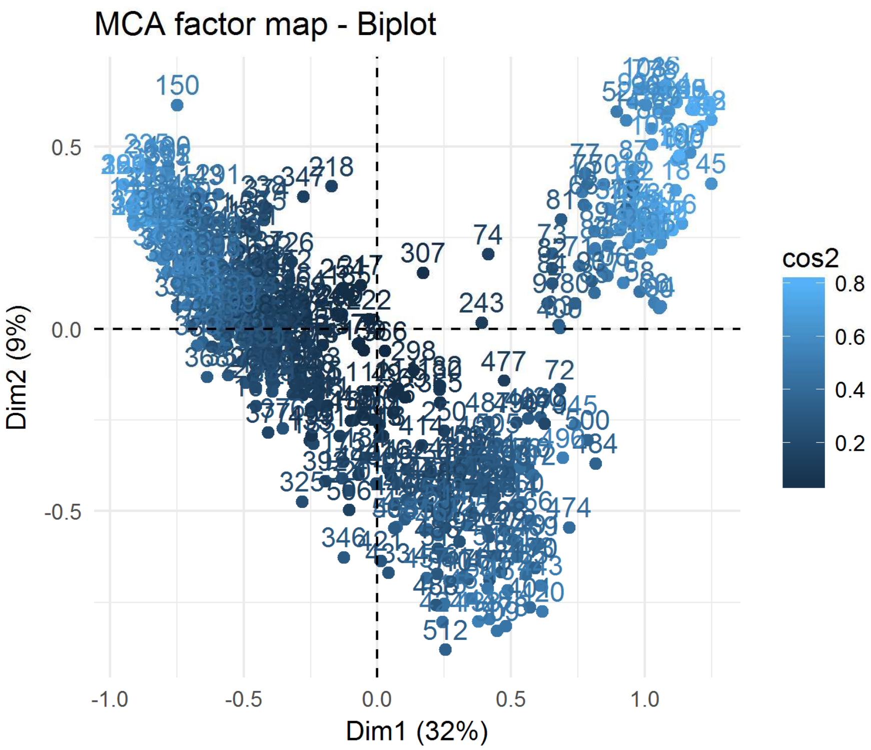

Figure A33. 1x2 dimension MCA factor map showing the squared cosine for each household.

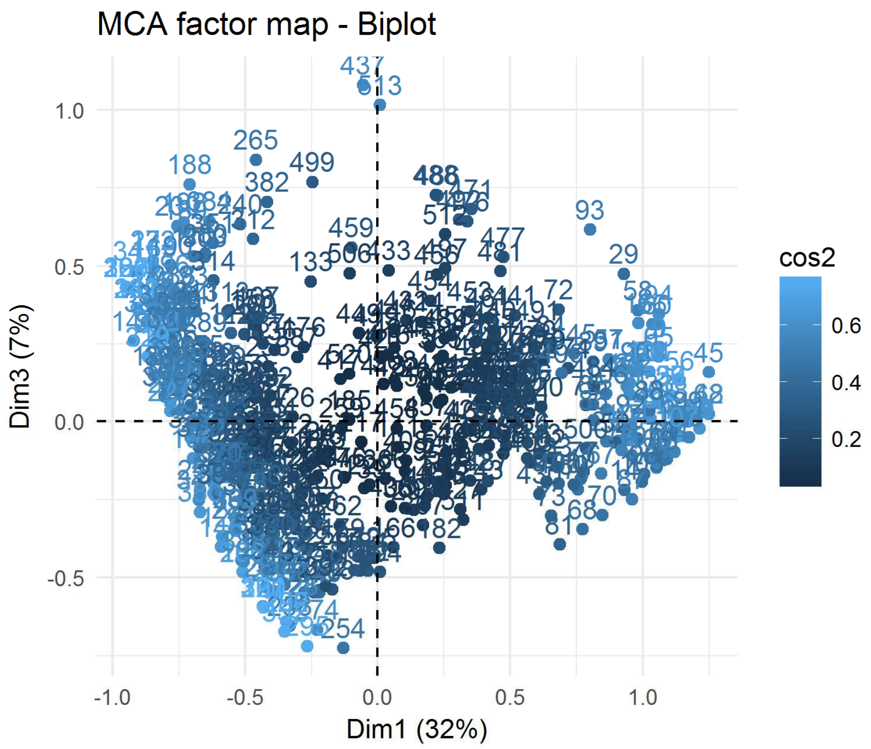

Figure A34. 1x3 dimension MCA factor map showing the squared cosine for each household.

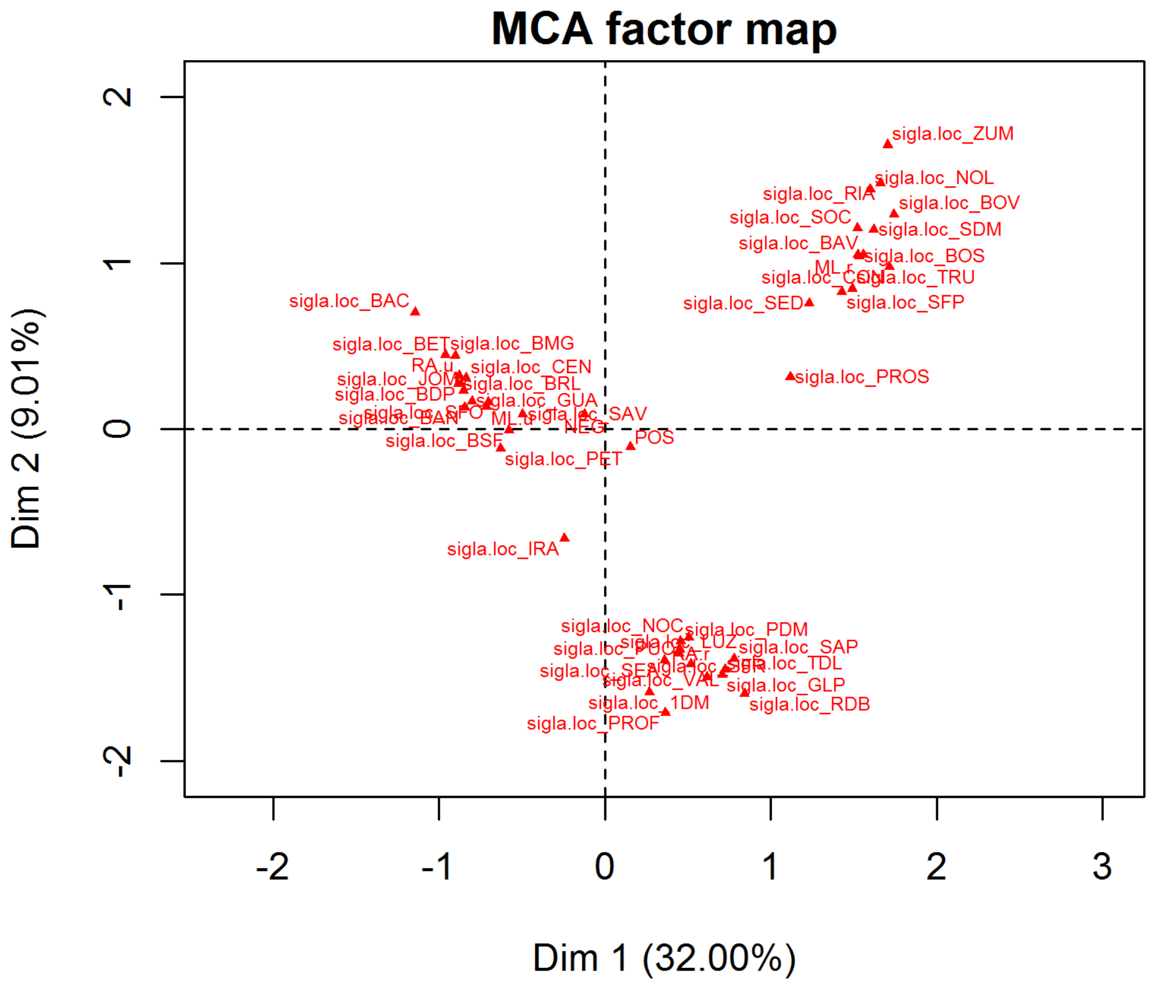

Figure A35. Distribution of the 40 localities in the 1x2 MCA plot. POS/NEG refers to the report of malaria in the last 12 months. These variables were included as Supplementary variables in the MCA analysis, so they did not participate in the MCA construction. See Additional file 1 for information on variable names.

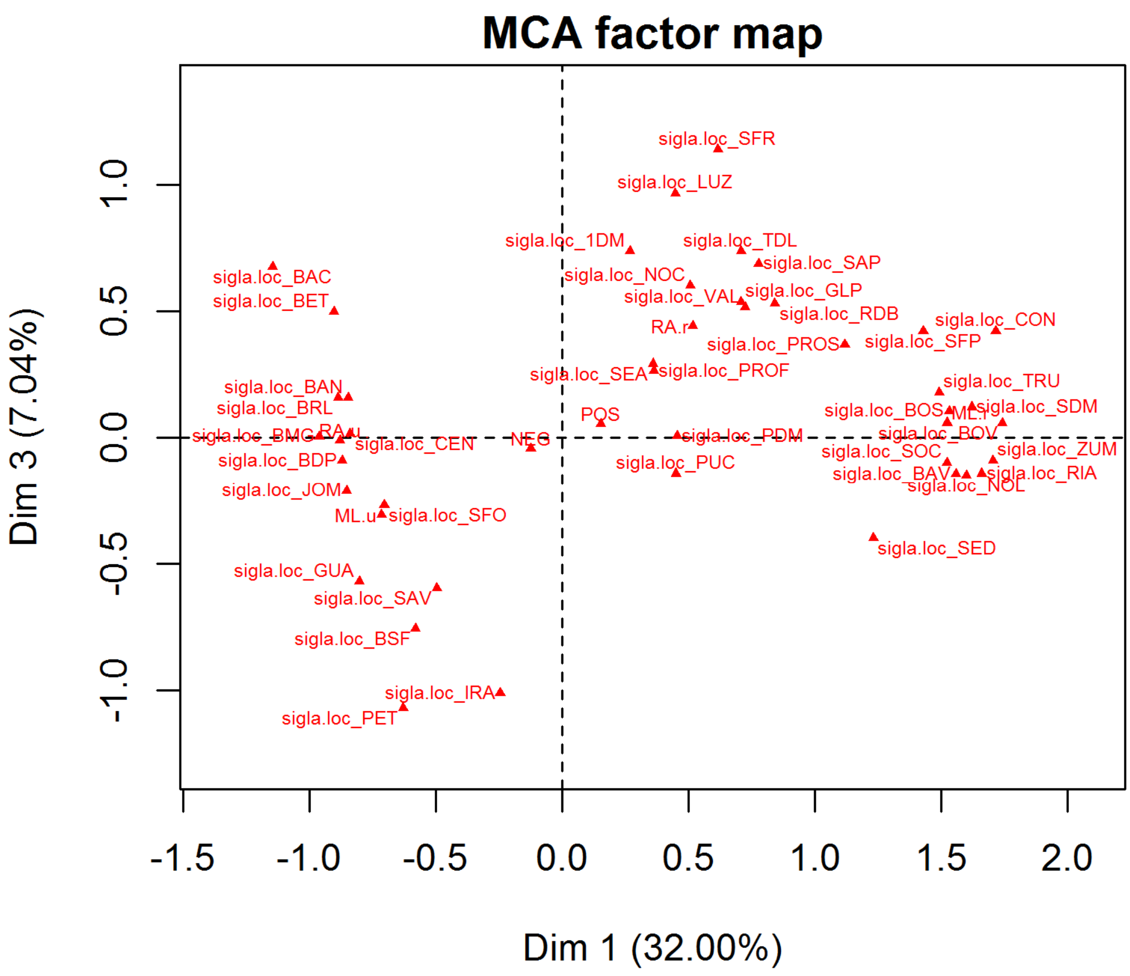

Figure A36. Distribution of the 40 localities in the 1x3 MCA plot. POS/NEG refers to the report of malaria in the last 12 months. These variables were included as Supplementary variables in the MCA analysis, so they did not participate in the MCA construction. See Additional file 1 for information on variable names.
