# Supplementary material for: Socioeconomic and demographic characterization of an endemic malaria region in Brazil by multiple correspondence analysis
Source: Malar J. 2017 Oct 2;16:397. doi: 10.1186/s12936-017-2045-z (PMC5625626; doi:10.1186/s12936-017-2045-z)
Supplement: Supplementary file 4 — Additional file 4. Details of mixed logistic regression models analyzes. [file 12936_2017_2045_MOESM4_ESM.pdf]

Additional file 4 - Details of Mixed Logistic Regression Models analyzes

Description: Mixed Logistic Regression Models for the outcome "self report of malaria in the household in the last 12 months".

The table shows the goodness-of-fit of the models with no fixed effects (Null) and models with the development gradients extracted from the MCA. Dim 1 = urban-rural gradient. Dim 2 = Rural-riverine gradient, Dim 3 = housing structure gradient. Zone refers to the model with a 4-level covariate (one for each study zone).

Model	Description	AIC	df
A	Null	698.423	2
B	Dim1 + Dim2 + Dim3	693.083	5
C	Dim1+ Dim2	692.368	4
D	Dim1	692.790	3
E	Zone	695.721	5

The figure below shows the marginal probability of malaria in households along the three development gradients. Each graph shows the effect of a given gradient setting the other two dimensions to 0.

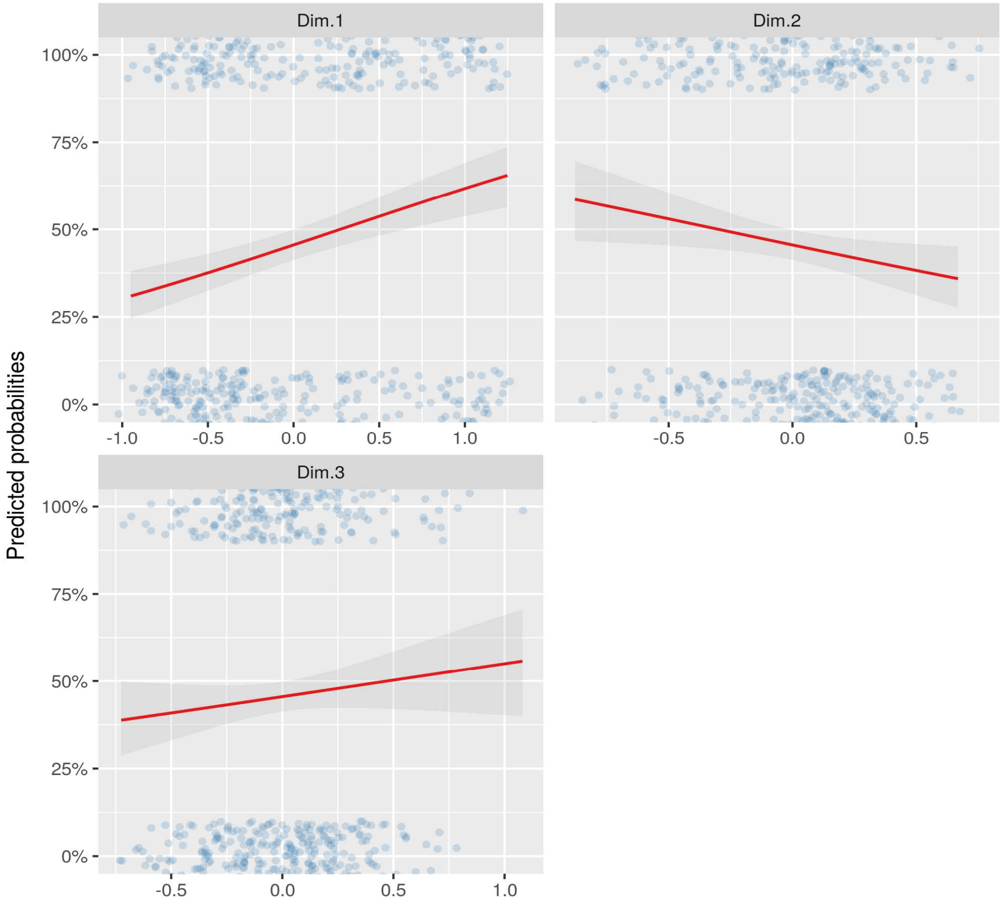

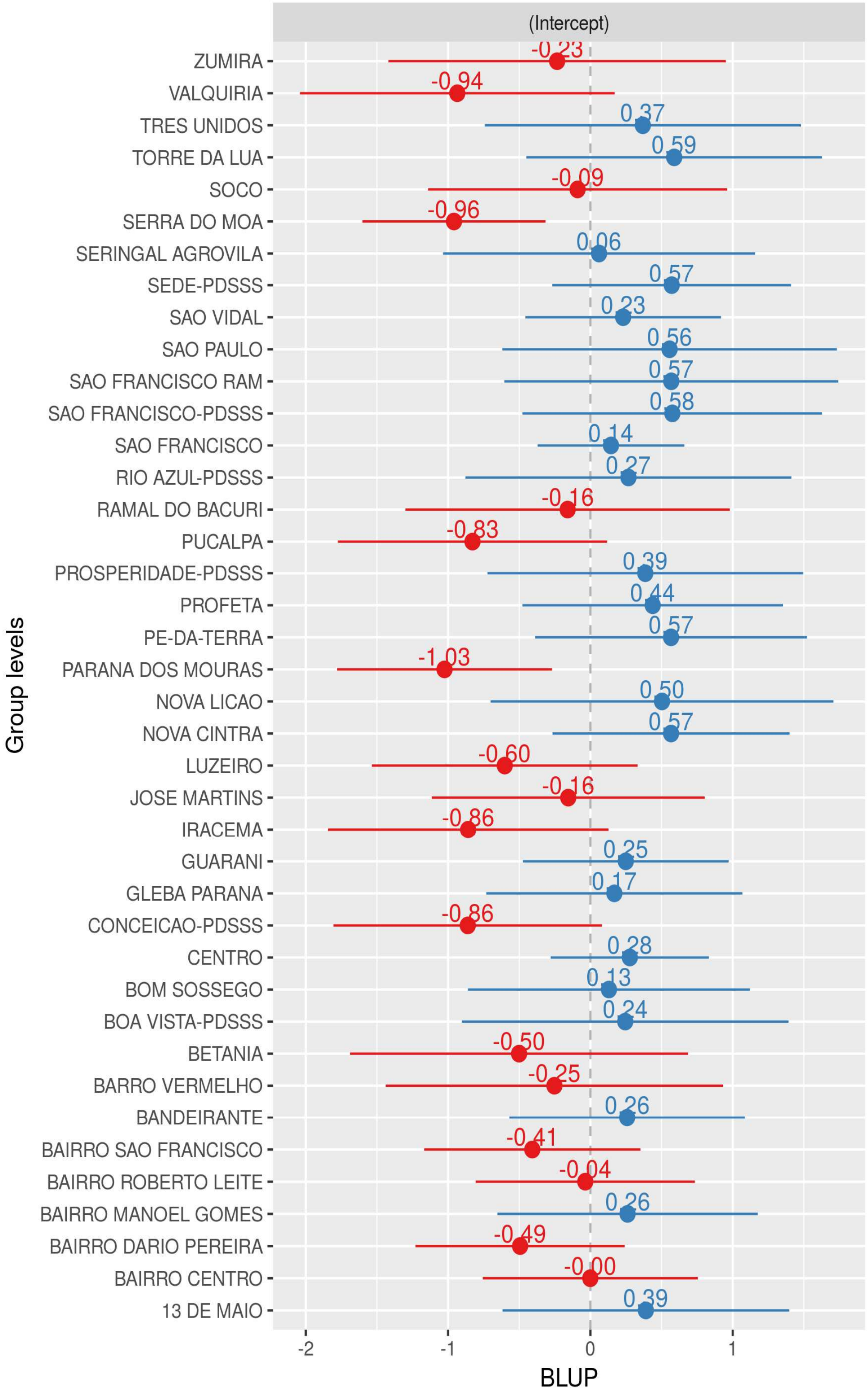

(Intercept)
ZUMIRA -0.23
VALQUIRIA -0.94
TRES UNIDOS 0.37
TORRE DA LUA 0.59
SOCO -0.09
SERRA DO MOA -0.96
SERINGAL AGROVILA 0.06
SEDE-PDSSS 0.57
SAO VIDAL 0.23
SAO PAULO 0.56
SAO FRANCISCO RAM 0.57
SAO FRANCISCO-PDSSS 0.58
SAO FRANCISCO 0.14
RIO AZUL-PDSSS 0.27
RAMAL DO BACURI -0.16
PUCALPA -0.83
PROSPERIDADE-PDSSS 0.39
PROFETA 0.44
PE-DA-TERRA 0.57
PARANA DOS MOURAS -1.03
NOVA LICAO 0.50
NOVA CINTRA 0.57
LUZEIRO -0.60
JOSE MARTINS -0.16
IRACEMA -0.86
GUARANI 0.25
GLEBA PARANA 0.17
CONCEICAO-PDSSS -0.86
CENTRO 0.28
BOM SOSSEGO 0.13
BOA VISTA-PDSSS 0.24
BETANIA -0.50
BARRO VERMELHO -0.25
BANDEIRANTE 0.26
BAIRRO SAO FRANCISCO -0.41
BAIRRO ROBERTO LEITE -0.04
BAIRRO MANOEL GOMES 0.26
BAIRRO DARIO PEREIRA -0.49
BAIRRO CENTRO -0.00
13 DE MAIO 0.39
Group levels
-2
-1
0
1
BLUP
